# Tracking Mitochondrial Density and Positioning along a Growing Neuronal Process in Individual *C. elegans* Neuron Using a Long-Term Growth and Imaging Microfluidic Device

**DOI:** 10.1523/ENEURO.0360-20.2021

**Published:** 2021-07-02

**Authors:** Sudip Mondal, Jyoti Dubey, Anjali Awasthi, Guruprasad Reddy Sure, Amruta Vasudevan, Sandhya P. Koushika

**Affiliations:** 1 National Centre for Biological Sciences, Tata Institute of Fundamental Research, Bangalore, Karnataka 560065, India; 2 Department of Mechanical Engineering, The University of Texas at Austin, Austin, Texas 78712; 3 Department of Biological Sciences, Tata Institute of Fundamental Research, Mumbai, Maharashtra 400005, India; 4 Institute for Stem Cell Science and Regenerative Medicine, Bangalore, Karnataka 560065, India; 5 Department of Biological Sciences, Birla Institute of Technology and Science, Pilani, Rajasthan 333031, India; 6 Sastra University, Thirumalaisamudram, Tamil Nadu 613401, India; 7 Manipal Academy of Higher Education, Manipal, Karnataka 576104, India

**Keywords:** C. elegans, touch receptor neurons, mitochondria, intermitochondrial distances, development, microfluidic device, long-term imaging

## Abstract

The long cellular architecture of neurons requires regulation in part through transport and anchoring events to distribute intracellular organelles. During development, cellular and subcellular events such as organelle additions and their recruitment at specific sites on the growing axons occur over different time scales and often show interanimal variability thus making it difficult to identify specific phenomena in population averages. To measure the variability in subcellular events such as organelle positions, we developed a microfluidic device to feed and immobilize *Caenorhabditis elegans* for high-resolution imaging over several days. The microfluidic device enabled long-term imaging of individual animals and allowed us to investigate organelle density using mitochondria as a testbed in a growing neuronal process *in vivo*. Subcellular imaging of an individual neuron in multiple animals, over 36 h in our microfluidic device, shows the addition of new mitochondria along the neuronal process and an increase in the accumulation of synaptic vesicles (SVs) at synapses. Long-term imaging of individual *C. elegans* touch receptor neurons (TRNs) shows that the addition of new mitochondria takes place along the entire neuronal process length at a rate of ∼0.6 mitochondria/h. The threshold for the addition of a new mitochondrion occurs when the average separation between the two preexisting mitochondria exceeds 24 μm. Our assay provides a new opportunity to move beyond simple observations obtained from *in vitro* assays to allow the discovery of genes that regulate positioning of mitochondria in neurons.

## Visual Abstract

**Figure:**
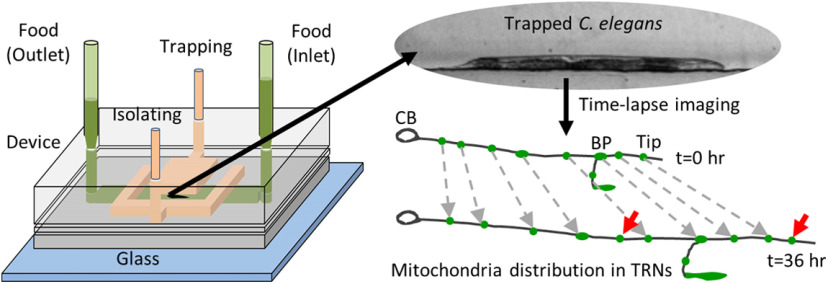

## Significance Statement

Axonal transport of mitochondria is required for the normal function and health of a developing animal with continuously growing axonal processes. Existing technologies are unable to monitor the addition of a new mitochondrion in a growing axon *in vivo*, as it requires continuous or intermittent tracking of the same individual neuron over several hours to days. We have developed a microfluidic device that enables long-term high-resolution imaging of individual *Caenorhabditis elegans* in an anesthetic-free setting. Using this device, we observe that the addition of a new mitochondrion can occur anywhere along the entire neuronal process, likely mediated by actively transported mitochondria, and at docking sites that occur with high probability when the separation between adjacent mitochondria crosses 24 μm threshold.

## Introduction

Development and maturation of neurons occur through the processes of cell birth, cell differentiation, cell migration, neuronal process outgrowth, dendritic arborization, synaptic growth, and organelle transport (Hobert, 2010; Smith et al., 2010; Varier and Kaiser, 2011; Smith and Gallo, 2018). Complex cellular architectures present in polarized cells such as neurons need the transport of organelles to support many energy-dependent processes such as neurotransmission (Sheng and Cai, 2012). One such organelle is mitochondria that are essential for neuronal function and thought to contribute to various disease pathologies (Liu et al., 2012; Millecamps and Julien, 2013). Only ∼20% of a large number of mitochondria in a neuronal process move and their halting depend on molecular motors, calcium, and other signaling molecules (Kang et al., 2008; Chen and Sheng, 2013; Schwarz, 2013; Sun et al., 2013; Misgeld and Schwarz, 2017). Mitochondrial trafficking continues throughout development when neurons undergo elongation over several fold and offers a unique opportunity to demonstrate the utility of long-term imaging in individual neurons. Time-lapse imaging of rapidly transported mitochondria in neuronal cultures over a few hours has identified that moving mitochondria preferentially dock between pairs of preexisting docked mitochondria (Miller and Sheetz, 2004) and thus help maintain a uniform mitochondrial distribution along axons (Miller and Sheetz, 2004; Misko et al., 2012). Thus far, many invertebrate axons have not typically been reported to have general anatomic features such as nodes of Ranvier enriched in mitochondria as observed in vertebrate neurons (Chiu, 2011; Ohno et al., 2011). Multiple neurons, both *in vivo* and in non-myelinated neurons in culture, show that the density of mitochondria is invariant (Wang and Schwarz, 2009a; Morsci et al., 2016; Misgeld and Schwarz, 2017; Sure et al., 2018). The majority of mitochondria in *Caenorhabditis elegans* touch receptor neurons (TRNs) have been reported to be present at actin-rich regions (Sood et al., 2018) and the density of mitochondria in adult animals remains largely invariant (Morsci et al., 2016; Sure et al., 2018). Despite averages being tightly regulated, interanimal variability is shown to exist even in nearly invariant cell lineages (Keil et al., 2017). Developing and aging neurons show variability in mitochondrial density and axonal length in a clonal genetic population (O’Toole et al., 2008), show heterogeneity of mitochondrial dynamics within individual cells (Sison et al., 2017), and show differential mitochondrial dynamics and distribution within different neuron segments (Obashi and Okabe, 2013). To investigate where mitochondria are added *in vivo*, one needs to analyze each mitochondrion along the entire neuronal process length, which can elongate significantly, i.e., >5-fold over ∼3 d in the same *C. elegans* animal.

Developmental events can be easily tracked in *C. elegans* using a microfluidic platform. Several microfluidic platforms have been developed for *C. elegans* to carry out high-resolution imaging (Chalasani et al., 2007; Mondal et al., 2011, 2012; Cáceres et al., 2012), high-throughput screening (Chung et al., 2008; Mondal et al., 2016, 2018), long-term imaging (Hulme et al., 2010; Krajniak and Lu, 2010; Xian et al., 2013; Keil et al., 2017; Atakan et al., 2019), and neuronal axotomy (Guo et al., 2008; Samara et al., 2010). Microfluidic device immobilization avoids alterations in *C. elegans* physiological processes caused by anesthetic immobilization (Guo et al., 2008; Mondal et al., 2011, 2012; Mondal and Koushika, 2014). Anesthetic-free immobilization can prevent mechanical stress induced by chemical immobilization methods that change muscle tone and body stiffness, leading to artificial elongation or contraction of the overall body length (Petzold et al., 2011). *C. elegans* can also be cultured by feeding them with bacterial cultures inside microfluidic devices (Krajniak and Lu, 2010; Krajniak et al., 2013; Xian et al., 2013; Lee et al., 2014; Gokce et al., 2017; Atakan et al., 2019). Microfluidic devices allow time-lapse neuronal imaging in well-fed animals for up to 24 h (Lee et al., 2014). In aging studies, long-term microfluidic devices have been used to grow *C. elegans* and acquire low-resolution images to quantify brood size and monitor locomotion (Xian et al., 2013; Rahman et al., 2018; Atakan et al., 2019). Recently, neuronal branching dynamics and lineage patterns were monitored using an imaging platform from an animal growing over 3 d (Keil et al., 2017). As yet, there is no study tracking subcellular events long-term such as monitoring organelle distributions or accumulations over time. To track cellular and subcellular events in identified individual animals, we developed a polydimethylsiloxane (PDMS) microfluidic device to grow *C. elegans*, completely immobilize animals in the same orientation, and image the same individual neuronal process throughout development.

Here, we describe the design and optimization of our microfluidic device and demonstrate its utility in capturing high-resolution fluorescence images of the same posterior lateral TRNs across 36 h of *C. elegans* development. Mitochondrial distribution along the neuronal process changes over time, however, the density of mitochondria was similar across different developmental stages. Our data suggest that maintenance of density likely occurs through the addition of a new mitochondrion between two mitochondria that are separated by at least 24 μm. Our device can also be used to study other events such as the accumulation of synaptic vesicles (SVs) at the synapses, occurring over several days.

## Materials and Methods

### Growth and imaging device fabrication

Our microfluidic device was fabricated using soft lithography of PDMS from SU8 resist features on silicon substrates (Whitesides et al., 2001). The device design consists of two photomasks designed in Clewin software and printed using a high-resolution laser plotter (Fine Line Imaging). The flow layer is comprised of a single 10-mm-long and 300-μm-wide straight channel with one inlet and one outlet reservoir. The control layer had two independently controlled membranes, through one central wide “trapping” channel and two narrow “isolating” channels. The trapping membrane is 2 mm wide to immobilize animals while the two interconnected isolation membranes are 300 μm wide to keep the worm within the restricted area of the flow channel. The circular pads for punches and fluidic connections are 2 mm in diameter. Two photomasks were used to produce SU8 masters using UV photolithography for the flow and control layers. The flow layer was fabricated using SU8-2025 (or SU8-2050) spun at 500 rpm for 5 s and 2000 rpm for 30 s to obtain thicknesses of ∼40 μm (or ∼80 μm). The control layer was created using SU8-2050 spun at 500 rpm for 5 s and 2000 rpm for 30 s to produce a thickness of ∼80 μm. PDMS (10:1) was prepared by mixing the PDMS base with the curing agent. The PDMS mix was spin coated on the flow layer at 1000 rpm for 35 s to produce ∼120 μm thickness on the SU8 master and baked at 70°C for 2 h. PDMS (10:1) was poured on the SU8 pattern for a 5-mm-thick PDMS mold for the control layer and baked at 70°C for 2 h. The baked control layer was cut and removed from the SU8 master. Access holes were punched for trapping and isolating channels and bonded on top of the flow layer using 18 W air plasma for 2 min. The bonded block was finally removed from the silicon substrate, holes were punched through for both inlet and outlet access, and bonded to a cover glass of thickness 170 μm.

### *C. elegans* strains

We used the *C. elegans* transgenic strains: *jsIs821* [*mec-7p::GFP::RAB-3*] (Kumar et al., 2010) and *jsIs609* [*mec-7p::MLS::GFP*] (Fatouros et al., 2012; Sure et al., 2018) in this study. The strain *jsIs821* expressed GFP::RAB-3, marking SVs of the six TRNs. For time-lapse mitochondria imaging, we used *jsIs609* strain expressing GFP targeted to the mitochondrial matrix in the six TRNs. Strains were maintained on NGM plates using standard protocols (Brenner, 1974). Eggs were transferred to fresh NGM plates and allowed to hatch for 2 h at 22°C. Long-term imaging and mitochondrial transport parameters were calculated at multiple time points up to 36 h after worms were placed in the device.

### *C. elegans* maintenance inside PDMS device

The flow layer of the device was filled with M9 buffer through the inlet and outlet. The two channels in the control layer were preconditioned with a buffer column under 14 psi pressure of nitrogen gas. When pressurized with compressed nitrogen gas, the column of liquid did not leak into the flow channel because of the presence of the PDMS membrane. A single egg or an early larval stage animal, growing on NGM plates, was pushed into the flow channel within the region between the two isolating channels. The two isolating membranes were turned on while the central trapping membrane was left free. The OP50 bacteria culture was diluted in S medium (1 × 10^8^ cells/ml), filled with 200 μl microtips, and connected to the inlet of the flow channel. The outlet was connected with the second pipette tip with a lower food volume compared with the first pipette tip at the inlet. The difference in the height of the food solutions connected to the inlet and outlet channels maintains a constant flow through the flow channel. The small overlap in an area between the isolating channels and the flow channel creates a partially closed membrane in the flow channel. The partially closed channel allowed bacteria to flow along the side of the walls of the flow channel while preventing the animal from crossing the isolating membranes. A wide central trapping membrane sealed the flow channel completely under similar gas pressure and immobilized animals completely for high-resolution imaging.

### Measuring body length and diameter

Individual animals growing inside the microfluidic devices and on NGM plates were imaged using a brightfield microscope (Olympus IX71) at 4× and 10× magnification. Images were loaded in Fiji (http://fiji.sc/Fiji) and individual worms, with dark features clearly visible on a light background, were traced with segmented lines. The segmented line was drawn from head to tail along the middle of the worm body to measure and quantify body length. The diameter was approximated with a straight line across the body in the vulva region as the maximum body width.

### Time-lapse imaging of *C. elegans* neurons

*C. elegans* growing on NGM plates were immobilized using either 3 mM levamisole on 2% agarose pads or without any anesthetic inside the microfluidic device under the trapping membrane for high-resolution fluorescence imaging. To count the total number of mitochondria at different developmental stages, we immobilized animals at different stages on an agarose pad using 3 mm sodium azide (Sigma-Aldrich) and imaged them within 10 min after the application of the anesthetic. Time-lapse fluorescence images were acquired using a 60× (oil immersion and the numerical aperture of 1.4) objective on an inverted microscope (Olympus IX81) equipped with a spinning disk unit (CSU Yokogawa) and a CCD-based camera (iXon, Andor) to visualize GFP fluorescence from *C. elegans* neurons. Long-term imaging during development was conducted on the same neuron using multiple images of the same neuron at different time points. To maintain animal physiology during long-term imaging experiments, animals were left free inside the flow channel in the presence of sufficient bacteria between successive imaging sessions. To estimate synapse growth, GFP::RAB-3 fluorescence was collected from the ventral synapses of the posterior lateral mechanosensory (PLM) neurons in device immobilized or 3 mM levamisole anesthetized animals at different stages of their development.

### Bleach and recovery of stationary mitochondria

*C. elegans* were mounted on a glass slide on a 2% agarose pad and anesthetized with 3 mM levamisole (Sigma-Aldrich) for live imaging. An Olympus IX83 inverted microscope attached with a spinning disk (PerkinElmer Ultraview) was used to acquire time-lapse images of the 60 μm length of the initial (first 1/3 from the cell body) or middle (2/3 from the cell body) of the PLM neuronal process at either 3 or 4 frames per second (fps) using a 60×, 1.4 NA oil immersion objective. The Andor (iXon DU897-UVB)/Hamamatsu (SZK) monochrome camera was used to capture transport movies from GFP-labeled mitochondria for 20 min using a 488-nm excitation laser at ∼8% transmission. At least 100 frames (25 s) of time-lapse movies were acquired to identify stationary mitochondria before bleaching one of the stationary mitochondria in the frame using a 405 nm laser at 60% transmission for 100 ms, 49 iterations of 10 ms each. Kymographs of the simultaneous bleaching and imaging movies were used to identify all the stationary bleached, stationary unbleached, and mobile mitochondria.

### Image analysis and quantitation

The physical dimensions of the *C. elegans* body were quantified using brightfield images of animals growing on NGM plates, in 96-well plate liquid cultures, and inside microfluidic devices. Fluorescence images were analyzed in Fiji. For mitochondrial density measurements, multiple image frames covering the entire TRN processes were opened using Fiji. Guided by the features and faint GFP signal present in the neuronal process, the length of the neuronal process was traced between successive mitochondria pairs using both the segmented and freehand line tools in Fiji. Because of linear geometry and absence of 3D curvature in the PLM neuronal process, both tools provided very similar length measurements. Hence, we used the segmented line tool for all our analyses. Every mitochondrial position and intermitochondrial intervals were recorded for every individual TRN at specified time points. Numerous total mitochondrial fluorescence envelopes were counted in a given neuronal process from the cell body to the distal tip of the process at each time point. Each fluorescence envelope was segmented to cover all the bright pixels to estimate the average intensity and total area for each mitochondrion. Intermitochondrial distances were quantified in pixels as distances between the centroid of two successive mitochondria in a neuronal process and converted to micrometers. For long-term mitochondrial analysis, the length of the neuronal process every 3 h was measured and plotted for an individual animal. A linear regression line was fitted to estimate the growth rate for each animal. Assuming a uniform growth rate, the length of the neuronal process at every 3 h time point was normalized to match the estimated value, the position of all mitochondria was corrected to match the total estimated neuronal process, and new intermitochondrial distances were calculated for further analyses.

Fast time-lapse fluorescence images of the mitochondria were converted to kymographs using the kymograph plugin in Fiji to quantify mitochondrial dynamics along the neuronal process. Kymographs were analyzed for stationary and moving mitochondria using line tracing. The anterograde and retrograde movements were determined using a minimum displacement cutoff of three pixels (0.6 μm) over five consecutive frames (1.67 s) and a velocity cutoff of >0.01 μm/s. Flux was calculated, using the total number of moving mitochondria normalized to the length of the movie and the entire visible neuronal process length. The fluorescence-decay profiles of stationary mitochondria were calculated by drawing an envelope around the stationary mitochondria before bleaching and the integrated intensity profiles at all successive frames prebleach and postbleach were quantified. To normalize variation in the background because of fluctuations in the laser intensity, we calculated the background signal from an area similar in size as the bleached mitochondrion within the worm but away from the neuronal process and subtracted it from the integrated values. The subtracted intensity profile was used to calculate the percentage drop and the photobleaching time for each stationary bleached mitochondrion. To calculate flux across bleached and unbleached mitochondria, anterogradely and retrogradely moving mitochondria were identified at locations with stationary mitochondria and normalized to the total time elapsed during each movie.

The size of SV accumulation at ventral synapses was calculated from the total number of pixels present inside the fluorescence envelope, consisting of all the pixels with fluorescence intensities higher than the background.

### Statistical analysis

Data are presented as the mean ± SD (or SEM). Histograms are used to show the intermitochondrial distances, gain insight into the shape and variability present in the data, and estimate probability distributions from the experimental values. We used the Freedman–Diaconis rule to estimate the bin size from the interquartile range (IQR) and the number of (n) intermitochondrial observations (Freedman and Diaconis, 1981). The bin width was estimated using $width=\frac{2\times IQR}{\sqrt[{3}]{n}}$, where the *IQR* is defined as the difference between the third and the first quartiles. To determine whether mitochondria are distributed uniformly, randomly, or clustered along the neuronal processes, we divided the neuronal processes into 20μm bins and counted the number of mitochondria in each bin. The number of mitochondria per bin, the SD of the mean, and variance (σ^2^) were calculated. We calculated the index of dispersion ($\frac{\sigma^{2}}{\mu }$) for mitochondrial distribution along the neuronal processes at *t* = 0 h (L4) and *t* = 24 h (D1) stage animals. Based on the index of dispersion, we determined whether mitochondria were distributed uniformly (μ > σ^2^), randomly (μ = σ^2^), or clustered (μ < σ^2^). To test the goodness of fit, we used the χ^2^ analysis against a Poisson distribution (Miller and Sheetz, 2004).

The normality for experimental observations was calculated using the Kolmogorov–Smirnov test and statistical significance was calculated using either the two-sample *t* test or one-way ANOVA (for more than two conditions). The *p < 0.05, **p < 0.005, and *p* > 0.5 (ns, not significant) was used to assess statistical significance.

## Results

### Mitochondrial number along the neuronal process increases during development in *C. elegans* TRNs

**Figure 1.**
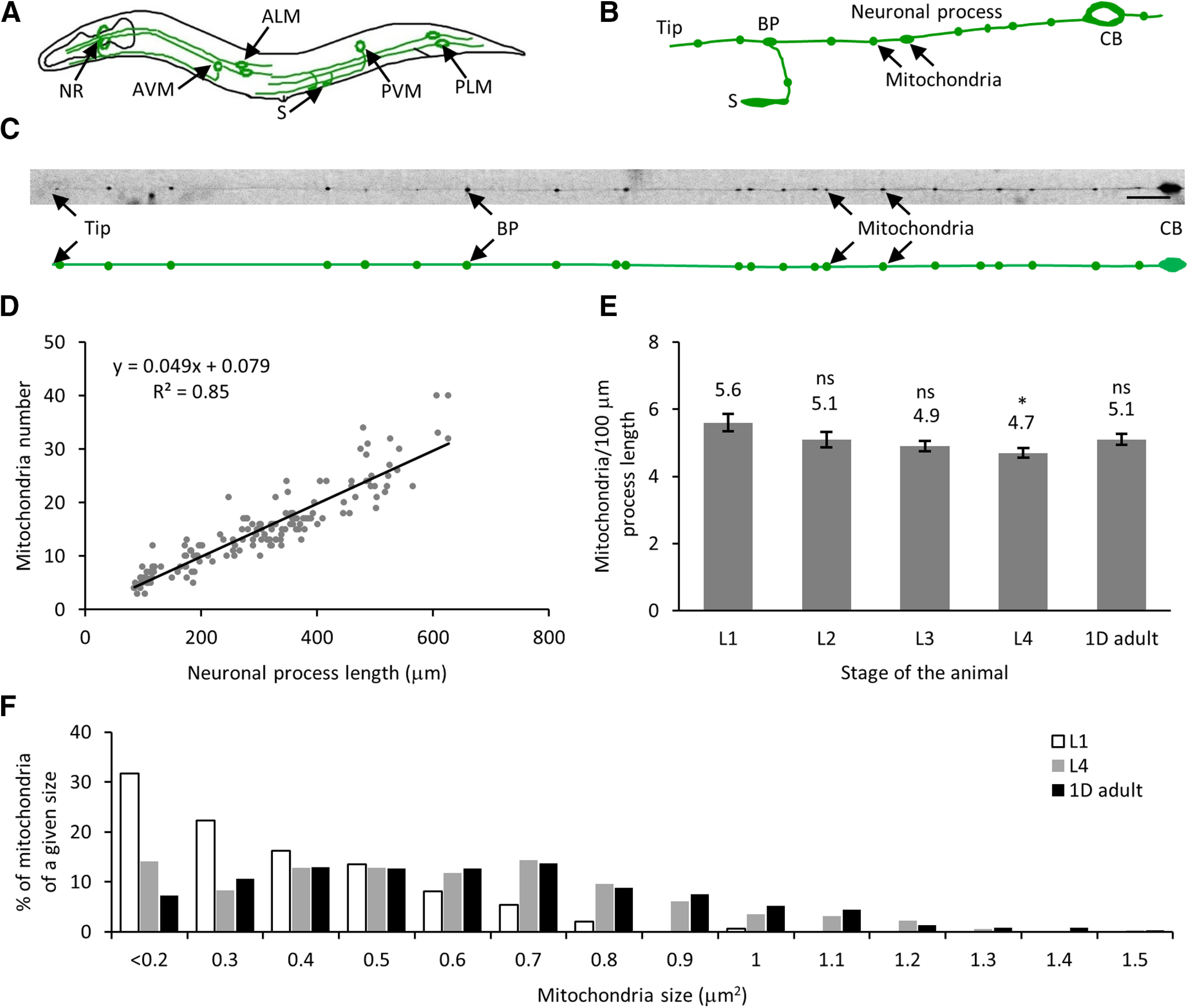
Mitochondria number and process length increase linearly with development. ***A***, Schematic of *C. elegans* with six TRNs. ***B***, Schematic of PLM neuron cell body (CB) with a long major neuronal process with a bifurcation of the process at the BP to form the synaptic branch that forms synapses (S) along the ventral cord. The mitochondria are indicated with arrows. ***C***, Image of a PLM neuron with 22 mitochondria along the neuronal process. The schematic below the image shows the positions of all 22 mitochondria in the same neuronal process. Scale bar: 20 μm. ***D***, Mitochondria number scales linearly with neuronal process length. Neuronal process length and number of mitochondria of all four larval stages (L1, L2, L3, and L4) and 1D adult. The trend follows a linear regression with a slope of 4.9 mitochondria in 100-μm neuronal process length (total number of animals = 154). ***E***, Mitochondria density remains constant throughout development. Data represented as mean ± SEM for L1 (*n* = 30), L2 (*n* = 25), L3 (*n* = 36), L4 (*n* = 33), and 1D adult (*n* = 30). Statistical significance was evaluated using one-way ANOVA with Bonferroni *post hoc* comparisons; *p < 0.05 and ns, *p* > 0.05 when compared with L1 stage. ***F***, Histogram of the normalized number of mitochondria of a given size represented as a % in the TRNs of L1, L4, and 1D adult stage of animals (*n* ≥ 148 mitochondria for each stage).

Mitochondria are essential organelles involved in energy metabolism and play a vital role in diverse biological processes such as aging and apoptosis (Sheng, 2014; Seervi and Xue, 2015; Sun et al., 2016; Melentijevic et al., 2017). Transport and distribution of mitochondria in neuronal processes and at synapses are critical for the normal physiology of neurons (Morsci et al., 2016; Cai and Tammineni, 2017). Since *C. elegans* are transparent throughout their development, it is feasible to track fluorescently labeled organelles such as mitochondria and SVs in TRNs using high-resolution imaging. *C. elegans* TRNs are a suitable model for high-resolution *in vivo* organelle imaging because of its long and planar neuronal process along the body length present close to the cuticle (Fig. 1*A*,*B*).

To quantify mitochondrial density in *C. elegans* TRNs, we immobilized *jsIs609* animals and imaged the TRNs using mitochondrial matrix targeted GFP expressed in six TRNs for a single time point analysis during their development (Fatouros et al., 2012; Sure et al., 2018). We immobilized the animals on a 2% agarose pad using 3 mm sodium azide and counted mitochondria number, within 10 min after the application of the anesthetic, using mitochondrial GFP and length of the neuronal process visible because of some GFP that fills the neuronal process in the PLM neurons (Fig. 1*C*). An increase in neuronal process length has been shown to correlate with an increase in mitochondria number as the animal ages and across developmental stages (Morsci et al., 2016; Awasthi et al., 2020). We found the neuronal process length increases from ∼100 μm in early larval stage 1 animals (L1) to nearly 500 μm in 1-d adult (1D adult) with a concomitant increase in mitochondria number from ∼6 to 26 in the posterior TRN (Fig. 1*D*). The bulk of the increase in the length of the neuronal process occurs as the animal grows and occurs after synapse formation at the early L1 stage of development. The addition of new mitochondria maintains a mitochondrial density of approximately five mitochondria per 100 μm along the neuronal process length throughout development (Fig. 1*E*; Table 1). This density is similar to earlier reports of density measured in adult animals for this neuron (Morsci et al., 2016; Sure et al., 2018). From the above values, the increase in mitochondrial number during development is approximated to approximately six mitochondria every 24 h (averaging approximately one mitochondrion every 4 h). The area of mitochondria fluorescence in L1, L4, and 1D adult stages of the animal is measured to vary between 0.05–0.95, 0.05–1.66, and 0.05–2.14 μm^2^, respectively (Fig. 1*F*). Young larval animals have a greater number of smaller mitochondria compared with later larval stages or adult animals. Single time point images of *C. elegans* populations of different developmental stages indicate a steady increase in the total number of mitochondria but fail to indicate the location of such addition. To capture individual mitochondrion addition events such that a similar mitochondria density is maintained over development, the same neuronal process needs to be imaged over days under optimal growth and imaging conditions. Since mitochondria are susceptible to cellular stresses (Hill and Van Remmen, 2014; Jovaisaite et al., 2014; Labbadia et al., 2017), it is impossible to image the same animal by repeatedly anesthetizing it over a prolonged growth period. To reduce the adverse effects of anesthetics and provide a physiological environment, we developed a long-term growth and imaging microfluidic platform.

**Table 1:** Statistical analysis for different figures

Figure	Normality	Test used	*p* value	Power
Fig. 1*E*	Yes	One-way ANOVA with Bonferroni *post hoc*	1.000	0.772
			0.091	
			0.016	
			0.689	
				
Fig. 2*H*	Yes	Two-sample *t* test	0.621	0.075
			0.391	0.129
			<0.005	1.000
			0.418	0.120
			0.027	0.645
			0.003	0.947
				
Fig. 2*I*	Yes	Two-sample *t* test	0.021	0.658
			0.012	0.779
			0.083	0.414
			0.012	0.778
			0.009	0.815
			0.143	0.303
				
Fig. 3*D*	No	Two-sample *t* test	0.06	0.433
			0.06	0.403
				
Fig. 3*E*	No	Two-sample *t* test	0.01	0.928
			0.14	0.348
				
Fig. 3*F*	No	Two-sample *t* test	0.20	0.239
			0.10	0.355
				
Fig. 3*G*	Yes	One-way ANOVA with Bonferroni *post hoc*	0.004	0.0917
			0.01	
				
Fig. 3*I*	No	Two-sample *t* test	0.07	0.199
			0.02	0.466
			0.03	0.238
			<0.005	0.363
			<0.005	0.482
			0.04	0.271
				
Fig. 3*J*	No	Two-sample *t* test	<0.005	0.509
			<0.005	0.630
			<0.005	0.552
			0.002	0.457
			>0.05	0.145
			>0.05	0.086
				
Fig. 3*K*	Yes	Two-sample *t* test	0.15	0.290
			0.45	0.113
				
Fig. 3*L*	Yes	Two-sample *t* test	0.30	0.174
			0.01	0.683
				
Fig. 3*M*	Yes	Two-sample *t* test	0.07	0.447
			>0.05	0.380
				
Extended Data Fig. 3-2*H*	Yes	Two-sample *t* test	0.50	
			0.47	
				
4*A*	Yes	Two-sample *t* test	<0.005	0.999
			<0.005	1
				
Fig. 4*B*	Yes	Two-sample *t* test	0.96	0.050
			0.13	0.321
				
Fig. 4*D*	Yes	Two-sample *t* test	0.92	0.051
				
Extended Data Fig. 4-2*A*	Yes	One-way ANOVA with *post hoc* Tukey	0.602	0.346
			0.606	
				
6*A*	Yes	Two-sample *t* test	0.02	0.692
			0.17	0.268
				
7*B*	Yes	One-way ANOVA with Bonferroni *post hoc*	1.0	1
			<0.005	
			<0.005	
			<0.005	
			<0.005	
			<0.005	
			0.74	
			1.0	
			0.01	
			0.18	

### Microfluidic device facilitates growth and high-resolution imaging of *C. elegans*

To study the long-term development of individual *C. elegans* while carrying out high-resolution imaging, we developed a microfluidic device to mimic a physiological environment by supplying constant food where we can immobilize the animal under a deformable PDMS membrane as needed. To make this microfluidic technology easily accessible, we developed a device with a simple design, few fabrication steps, easy operation, no complex valves, using inexpensive accessories. The PDMS device is bonded on a thin cover glass to facilitate high-resolution imaging on an inverted microscope using lenses of various magnifications up to a 100× oil objective.

**Figure 2.**
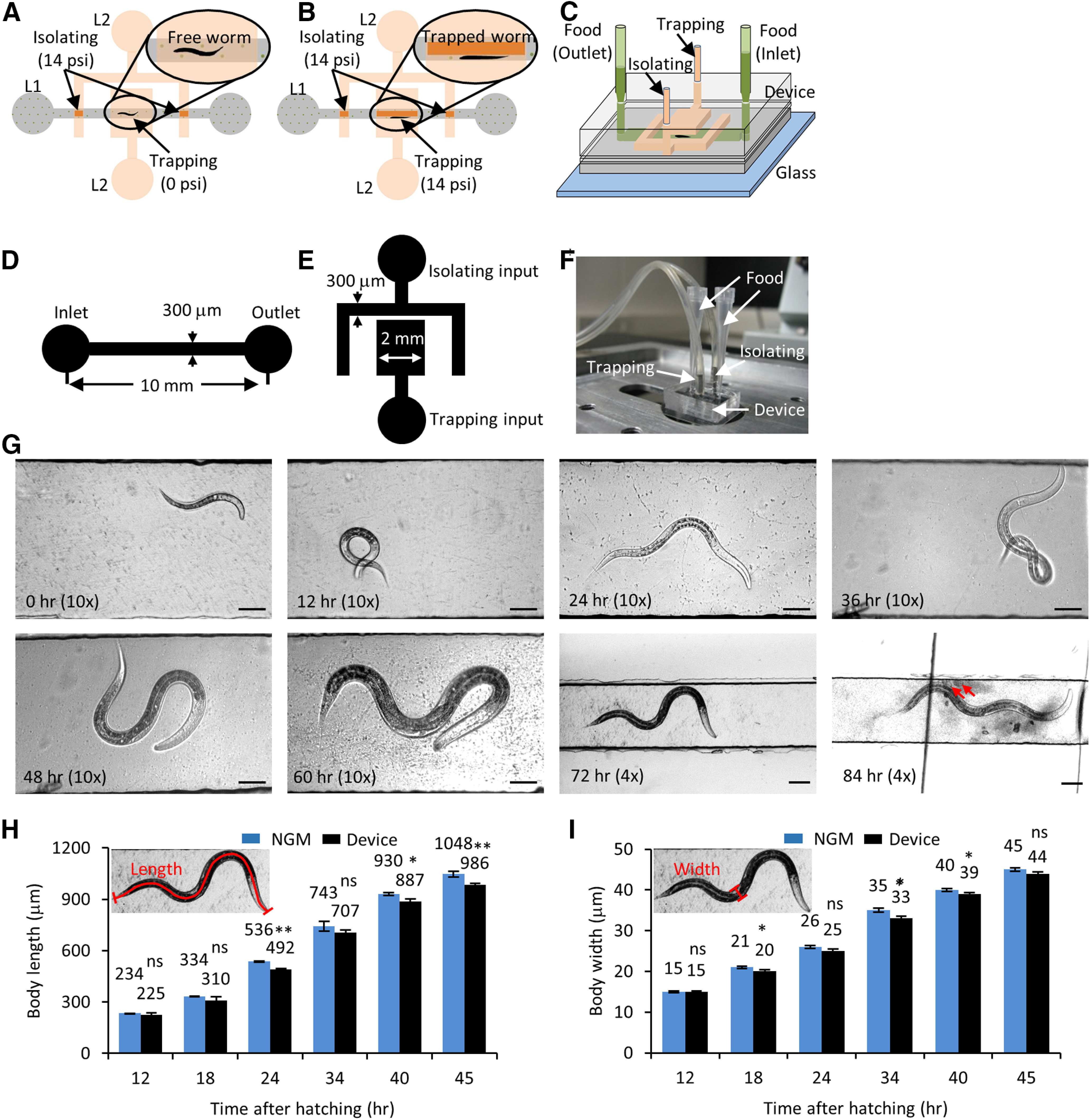
Growth and development of *C. elegans* are unaffected in the long-term growth and imaging microfluidic device. ***A***, Schematic of the microfluidic device with a flow channel in the bottom layer (L1, gray) and isolating and trapping channels in the control layer on top (L2, orange). The isolating membrane is always kept on, with 14 psi pressure (deflected membrane denoted by dark orange) to restrict the free animal shown in the inset. ***B***, Trap pressure is turned on (14 psi, deflected membrane denoted by dark orange) to immobilize the animal inside the flow channel (inset) during imaging. ***C***, 3D view of the device layout with two pipettes tips (light green) as food inlet and outlet connecting the flow channel (dark green). ***D***, Schematic for the flow layer with channel dimensions. ***E***, Schematic of the control layer with channel dimensions. ***F***, Image of a growth and imaging device connected to compressed nitrogen gas and food supply. ***G***, Images of a *C. elegans* hermaphrodite growing inside the microfluidic device at 0, 12, 24, 36, 48, 60, 72, and 84 h posthatching. The animal is fed OP50 bacteria and isolated inside the flow channel and imaged with a 4× or 10× objective. The red arrows indicate freshly laid eggs at 84 h. Scale bars: 50 or 100 μm at the 72- and 84-h time points. ***H***, ***I***, *jsIs609* animals grown in the device (black) and on regular NGM plates (blue) were used for calculating body length (***H***) and body diameter (***I***). The small inset shows the schematic for body length and body diameter from worm images. The average values are mentioned on top of each bar. Data represented as mean ± SEM, *n* = 12 animals. Statistical significance was evaluated by paired sample *t* test; *p < 0.05, **p < 0.005, and ns, *p* > 0.05 when compared between animals grown on NGM and in liquid culture.

The device is fabricated as two separate PDMS layers and irreversibly bonded together (Fig. 2*A–F*). The device utilizes PDMS membrane deflections to isolate and immobilize a *C. elegans* hermaphrodite within the flow channel. In this device, *C. elegans* swim freely and grow in a 300-μm-wide flow channel in the presence of bacteria. A pair of partially closed membranes above the symmetric isolating channels allows the bacterial suspension to pass through while preventing an individual animal from escaping the flow channel. The individual animal is repeatedly immobilized under a deflected “immobilizing” PDMS membrane. *In vivo* cellular processes are imaged in the animal immobilized beneath the trapping channel.

### Growth in a microfluidic device does not impede *C. elegans* development

To obtain high-quality time-lapse movies of the subcellular events during *C. elegans* development, the health of the animal grown in a microfluidic environment during the entire period should be similar to that of an animal grown on standard NGM solid media. *C. elegans* growth in liquid medium with *Escherichia coli* bacteria has been studied (Solis and Petrascheck, 2011; Keil et al., 2017). *C. elegans* were found to develop ∼15% slower in liquid culture with adequate bacterial supply nonetheless recapitulated all long-term developmental processes such as molting and egg laying (Keil et al., 2017). Since food supply is one critical component for normal development, we ensured sufficient bacterial supply by adjusting both the concentration of bacteria and the relative amounts of buffer solution with bacteria in the two pipette tips attached to the channel entrances. The flow channel containing liquid OP50 showed no contamination over 84 h (4 d) of *C. elegans* growth (Fig. 2*G*). The pipette tips can be easily replaced with new sterile tips freshly filled with OP50 bacteria as needed.

To characterize *C. elegans* development in our microfluidic device, we measured the body length and diameter of the animals grown in our microfluidic chamber and compared them to values obtained from animals grown on NGM solid media. Our data show that animals growing in the microfluidic device are on average shorter and thinner compared with those grown on solid NGM media (Fig. 2*H*,*I*; Table 1). The body features (body length and diameter) of the animals grown inside the microfluidic device lags by 2–3 h compared with the values obtained from those grown on NGM solid media. These data are similar to those reported earlier for *C. elegans* grown in liquid media (Solis and Petrascheck, 2011; Keil et al., 2017). A hermaphrodite grown inside the microfluidic device laid eggs after 84 h, that hatch into larvae.

### Microfluidic device facilitates repeated immobilization and time-lapse imaging of the identical neuronal process in an individual animal

**Figure 3.**
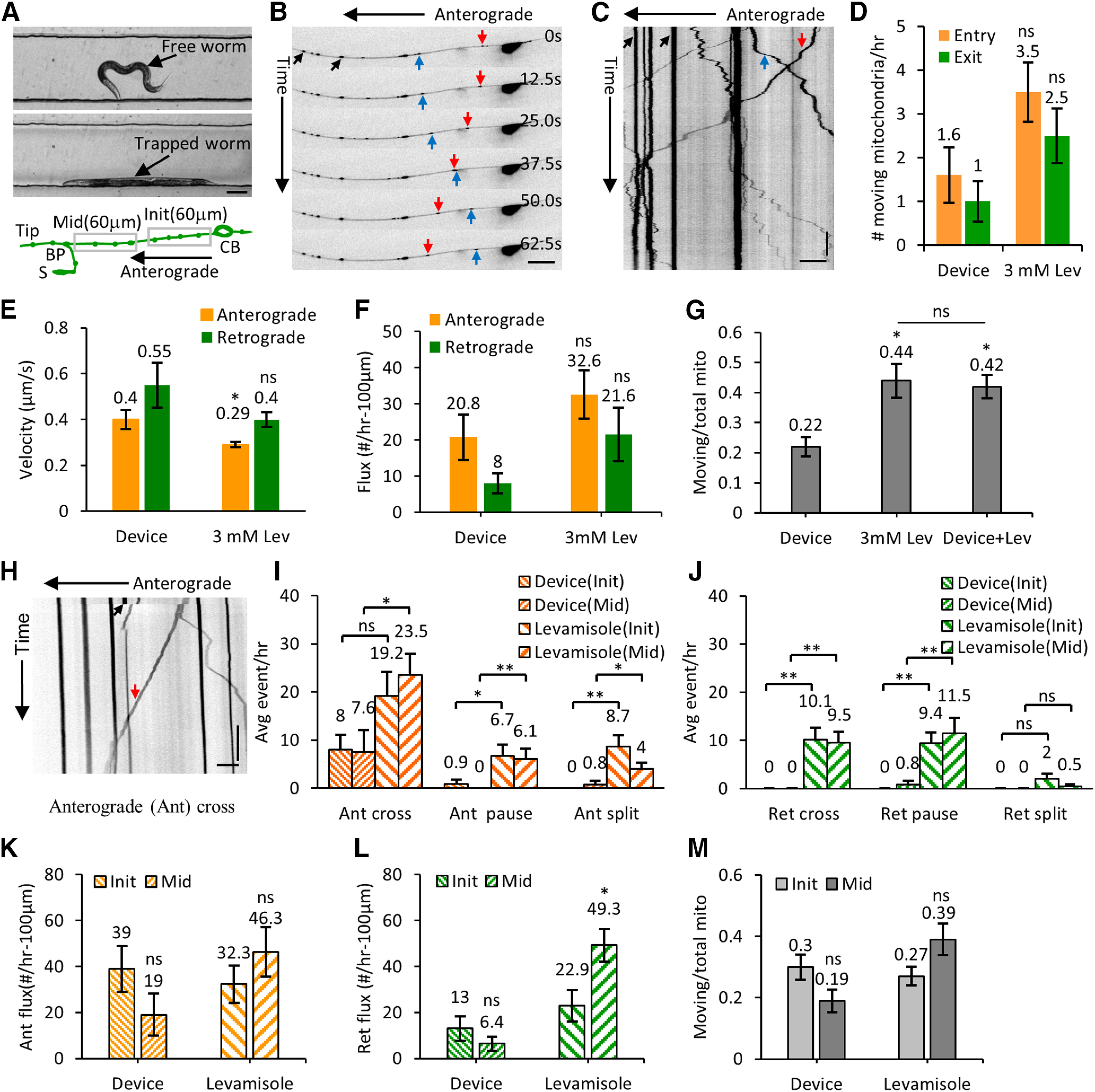
High-resolution transport imaging of GFP-labeled mitochondria in levamisole and device immobilized *C. elegans* neurons. ***A***, Image of a free and an immobilized L4 stage *C. elegans* in the flow channel. The schematic shows the initial 60 μm from the cell body and the middle of the neuronal process (120 μm from the BP toward the cell body) that is imaged at high resolution. ***B***, Montage of six frames of a PLM neuron with GFP-labeled mitochondrial fluorescence from time-lapse imaging acquired using a 60× oil objective (1.4 NA). ***C***, Kymograph of 400 frames acquired at 2 fps for 200 s. Anterogradely moving (red arrow), retrogradely moving (blue arrow), and stationary (black arrow) mitochondria are indicated on the images. ***D***, Number of mitochondria entering the neuronal process (entry) and leaving the process (exit) per hour measured in animals immobilized with 3 mM levamisole (*n* = 35 animals) and inside the microfluidic device (*n* = 22 animals). ***E***, The average velocities (both in anterograde and retrograde directions) are shown for animals that are immobilized inside the microfluidic device (*n* = 47 anterograde and *n* = 15 retrograde segments) and compared with animals immobilized with 3 mM levamisole (*n* = 336 anterograde and *n* = 142 retrograde segments). ***F***, The average flux values of moving mitochondria from L4 stage animals immobilized inside the microfluidic device (*n* = 16) and with 3 mM levamisole (*n* = 18). ***G***, The plot showing the ratio of moving mitochondria to the total number of mitochondria for device immobilized (*n* = 16), levamisole immobilized (*n* = 18), and levamisole treated animals that are also immobilized in the device (*n* = 14). ***H***, Kymograph representing an anterograde moving mitochondrion (red arrow) crossing a site of a bleached stationary mitochondrion (black arrow). ***I***, The average anterograde events per hour (cross, pause, and split) are measured at the initial (Init; 60 μm from the cell body) and the middle (Mid; a region 120 μm from the BP toward the cell body) of the neuronal process (schematic is shown in panel ***A***) for both device (Device; *n* > 8 animals) and anesthetic (Levamisole; *n* > 29 animals) immobilized L4 animals. ***J***, The average retrograde events are measured by using the device and anesthetic immobilized animals. ***K***, The anterograde flux are measured from all the moving mitochondria in the device and levamisole immobilized L4 animals from the Init and Mid of the PLM neuronal process. ***L***, The retrograde flux are measured from all the moving mitochondria in the device and levamisole immobilized L4 animals from Init and Mid neuronal processes. Anterograde and retrograde fluxes are calculated from *n* = 10 (Init) and *n* = 10 (Mid) animals in the device, while *n* = 16 (Init) and *n* = 26 (Mid) animals in the levamisole. ***M***, The ratio of moving mitochondria to the total number of mitochondria for the Init and Mid portion of the PLM in the device and levamisole immobilized animals. Data represented as mean ± SEM (***D***, ***E–G***, ***I***–***M***). Statistical significances are evaluated by paired sample *t* test (***D–F***, ***I–M***) and one-way ANOVA (***G***); *p < 0.05, **p < 0.005, and ns, *p* > 0.05 are represented. Scale bars: 100 μm (***A***), 10 μm (***B***, ***H***), and 5 μm (***C***). Vertical bars: 20 s (***C***, ***H***). Table 2, Extended Data Figures 3-1, 3-2, and Movies 1, 2, 3 support this figure.

Mitochondria in the neuronal process display distinct transport characteristics; characteristic velocities away from (anterograde) and toward (retrograde) the cell body, intermittent pauses, and bi-directional movement (Welte, 2004; O’Toole et al., 2008; Cai and Sheng, 2009; Schwarz, 2013; Hancock, 2014; Sure et al., 2018). To quantify *in vivo* mitochondrial transport characteristics from the *C. elegans* TRN processes, we immobilized animals in the flow channel using 14 psi on the trapping PDMS membrane (Fig. 3*A*). A slow progressive immobilization allows animals to be laterally oriented, facilitating high-resolution imaging of PLM neurons.

To assess the effect of photobleaching and changes in auto-fluorescence because of repeated immobilization, we repeatedly immobilized *C. elegans* at two different time intervals; either trapping every 1 h or trapping every 6 h under the immobilizing membrane up to a total duration of 12 h. Under both paradigms, the same PLM neuron (either L or R) of the animal was imaged only at the 0-, 6-, and 12-h time points. The ratio of the mitochondrial fluorescence to the background fluorescence (M/B ratio) was calculated at different time points. The decrease in this fluorescence ratio in both paradigms was statistically insignificant (2.07 ± 0.106, 1.80 ± 0.192, *p *=* *0.06; Extended Data Fig. 3-1*A–C*). Using repeated imaging of *C. elegans* in our microfluidic device, we were able to obtain a good fluorescence signal from mitochondria compared with the background autofluorescence.

Individual animals of the appropriate developmental stage were picked from an NGM plate, using 5 μl M9 buffer, and inserted inside the flow channel in the absence of immobilization or isolation pressure. The animal inside the flow channel was observed using either a 4× or a 10× objective on an inverted microscope. The height of the bacterial solution in the two pipette tips, connected at the inlet and outlet, generated sufficient hydrodynamic flow rates to allow bacterial passage through the flow channel to feed the worm (Fig. 2*C*,*F*). The deflected membrane in the isolating channel restricts animal motion between the two isolating channels. Freely moving worms were imaged in brightfield to measure their physical parameters and assess animal health. Animals were completely immobilized in the flow channel under the PDMS membrane using 14 psi pressure in the trapping channel. Complete immobilization was required for high-resolution time-lapse imaging of the subcellular events in the neuronal processes. Animals were immobilized repeatedly in a straight posture along the flow channel sidewalls under the trapping membrane to bring the same posterior lateral TRN under the best focus for imaging the subcellular events (Fig. 3*A*). Identical animal orientation enabled us to image mitochondrial distribution from the same individual neuron over multiple time points using an inverted confocal microscope equipped with a 60× oil objective (numerical aperture 1.4).

10.1523/ENEURO.0360-20.2021.f3-1 Extended Data Figure 3-1 Effect of photobleaching and intermitochondrial distances during long-term mitochondrial imaging. ***A***, Schematic of the TRN neuron. The inset shows a single mitochondrion and a 5 × 5 μm box to calculate the average intensity values for a mitochondrion (M) and the background (B). ***B***, Image of a single neuron at zero time shows a single mitochondrion (M) and the background box (B) on the worm body. Scale bar: 10 μm. ***C***, Fluorescence ratio of the mitochondria intensity to background intensity (M/B) calculated from the images of the same animal captured at 0th, 6th, and 12th hour imaging time points. The animals were immobilized at two different time intervals of every 1 h and every 6 h, respectively. Time-lapse imaging of the same animal over 12 h shows statistically insignificant photobleaching of mitochondrial fluorescence (*n* = 4 animals, 5 mitochondria, and a box drawn around each mitochondrion was used to calculate the statistics). Data represented as mean ± SD. Statistical significance was evaluated by one-way ANOVA with Bonferroni *post hoc* comparisons; nonsignificant values (*p* > 0.05) are not indicated. ***D***, Number of events for the percentage of compression or expansion measured from relative intermitochondrial distances (ΔL/L, *n* =* *292 events). The inset shows a pair of stationary mitochondria (M_1_ and M_2_) with an intermitochondrial distance of L compressed to L-ΔL. ***E***, Average compression (M_1_ and M_2_ appear closer) or expansion (M_1_ and M_2_ move further apart) percentage values. The data represented as mean ± SEM (*n* =* *8 animals imaged for 3 successive time points at 5 min intervals). Download Figure 3-1, TIF file.

10.1523/ENEURO.0360-20.2021.f3-2 Extended Data Figure 3-2 Kymographs of levamisole immobilized L4 *C. elegans* that show bleaching of stationary mitochondria and anterogradely moving mitochondria across the bleached mitochondria. ***A***, Kymograph of stationary mitochondria (red up arrow) that was bleached at 41.0 s (horizontal blue arrow) in the PLM neuronal process. Horizontal and vertical scale bars: 20 μm and 33 ms, respectively. **B**, The box and whisker plot shows the percentage drop in the integrated mitochondrial fluorescence intensity during photobleaching of *n* = 20 stationary mitochondria. The plot shows the median line, mean marker (×), and the outlier (o). **C**, The fluorescence intensity profile of stationary mitochondria that was bleached using a 405-nm laser. The arrow indicates the time when each of the three mitochondria were bleached at 29.0, 37.0, and 41.0 s. ***D*–*F***, Anterogradely moving mitochondria (red down arrow) crosses (***D***), pauses (***E***), and partially splits (***F***) at the site of the bleached mitochondria (red up arrow). The blue horizontal line indicates the bleaching of the stationary mitochondrion. Scale bar: 10 μm. ***G***, The intensity distributions along the neuronal process length and at five different time points (T_0_, T_1_, T_2_, T_3_, and T_4_), represented by the red dotted lines in ***F***. ***H***, The average flux of moving mitochondria across a bleached (*n* = 15) and an unbleached (*n* = 18) mitochondrion. The data represented as mean ± SEM. Statistical significance was evaluated by paired sample *t* test; nonsignificant values (ns, *p* > 0.05) are represented. Download Figure 3-2, TIF file.

Movie 1. Time-lapse movie showing a cross event across a bleached stationary mitochondrion indicated by the arrow. 10.1523/ENEURO.0360-20.2021.video.1

Movie 2. Time-lapse movie showing a pause at the location of a bleached stationary mitochondrion, indicated by the arrow. 10.1523/ENEURO.0360-20.2021.video.2

Movie 3. Time-lapse movie showing a split event at the location of a bleached stationary mitochondrion, indicated by the arrow. 10.1523/ENEURO.0360-20.2021.video.3

**Table 2:** Table representing the transport parameters and the turnover of mitochondria at stationary mitochondria from L4 animals anesthetized using the microfluidic device and 3 mM levamisole

	Device		3 mM levamisole	
	Init PLM	Mid PLM	Init PLM	Mid PLM
Anterogradely moving cross	83%	66%	36%	43%
Retrogradely moving cross	0%	0%	17%	18%
Anterogradely moving contributes to recovery	17%	17%	26%	18%
Retrogradely moving contributes to recovery	0%	17%	21%	21%
Total movies/total events analyzed	8/6	12/6	29 / 88	37 / 109
Anterograde velocity (Device)	0.40 ± 0.042 (*n* = 47)		0.29 ± 0.011 (*n* = 336)	
Retrograde velocity (Device)	0.55 ± 0.098 (*n* = 15)		0.40 ± 0.031 (*n* = 142)	

We further characterized the effect of immobilization of an individual *C. elegans* on the variability in the position of each stationary mitochondrion that arises just from the immobilization process and small changes in the posture of the animal. Positions of mitochondria were used to calculate intermitochondrial distances and estimate local compression and expansion of the neuronal process between multiple adjacent mitochondrial pairs. We, therefore, imaged the same TRNs every 5 min for 15 min by repeatedly trapping the animal under the immobilizing PDMS membrane and calculated intermitochondrial intervals for each immobilization. Intermitochondrial distance values (L) between identical mitochondrial pairs from the same TRN processes imaged at different time points were compared (ΔL). This comparison allows estimation of how the extent of expansion or compression between each mitochondria pair (ΔL/L) affects adjacent intermitochondrial distances. Only stationary bright mitochondria were considered for this calculation. Most mitochondrial positions remain largely unchanged and intermitochondrial distances were used to measure the compression or expansion percentages between each pair of mitochondria (Extended Data Fig. 3-1*D*,*E*). The compression and expansion percentages were distributed symmetrically and represented as positive (compression) and negative (expansion) values, respectively. The ΔL/L values were distributed around +1.79% and −2.01% (2.4 ± 0.17 and 2.4 ± 0.19 μm, mean ± SEM; Extended Data Fig. 3-1*E*). Thus, the errors arising from compression and expansion because of immobilization do not significantly alter the measurement of intermitochondrial distances.

### Device-immobilized animals show lower mitochondria flux and turnover compared with anesthetic immobilized animals

We examined mitochondria numbers and their dynamics in NGM grown and device grown animals. The density of mitochondria in larval stages and 1D adult animals grown in NGM and microfluidic devices are similar. Additionally, the intermitochondrial intervals in NGM grown animals and device grown animals are very similar. To examine the movement and turnover of mitochondria from both device and anesthetic immobilized animals, we conducted high-resolution time-lapse imaging of TRNs. The acquired movies were converted into kymographs to analyze mitochondrial transport and positions (Fig. 3*B*,*C*). Device immobilized animals regained normal body movements within 10 min of release from pressure. Mitochondria are classified as stationary if their displacement is less than three pixels (0.6 μm) over five consecutive frames (1.67 s) or their velocity is <0.01 μm/s. A mitochondrion is considered as moving if its displacement is more than or equal to three pixels over five consecutive frames. Mitochondria move anterogradely away from the cell body or retrogradely toward the cell body. The movement close to the cell body was captured for 20 min at 3 fps to quantify all anterograde and retrograde events in both anesthetized animals and device immobilized L4 stage animals, both grown on NGM plates until imaged. The average number of mitochondria entering the neuronal process from the cell body was about twice the total number of mitochondrial exit events into the cell body (Fig. 3*D*). The average number of entry and exit events was lower but not statistically different (*p* > 0.5) in the device immobilized animals compared with anesthetized animals (Table 1). In device immobilized animals, the rate of mitochondrial entry into the process is twice that of mitochondrial exit as well (Fig. 3*D*).

The average instantaneous velocity of moving mitochondria in both the anterograde and retrograde directions was slightly higher in the device (0.40 ± 0.042 and 0.55 ± 0.098 μm/s) compared with anesthetic immobilized animals (0.29 ± 0.011 and 0.40 ± 0.031 μm/s), although statistically significantly different only for the average anterograde velocities (Fig. 3*E*; Table 2). The average anterograde mitochondria flux value was higher than that of the retrograde flux in both device and anesthetic immobilized animals (Fig. 3*F*). The anterograde and retrograde fluxes in the levamisole immobilized animals (32.6 ± 6.70 and 21.6 ± 7.40 mitochondria/h/100 μm) were higher as compared with that in the device immobilized animals (20.8 ± 6.28 and 8.0 ± 2.74 mitochondria/h/100 μm) although the differences are not statistically significant (*p* = 0.20 and *p* = 0.10). We examined whether the greater mitochondrial transport in levamisole (3 mM Lev) treated worms, compared with worms immobilized in the device without chemicals (Device) was because of the anesthetic. We acquired time-lapse images of PLM neurons from animals both anesthetized in 3 mM levamisole and trapped in our microfluidic device (Device + Lev). The ratio of all moving mitochondria to the total number of mitochondria in levamisole immobilized (0.44 ± 0.056, *n* = 18) animals were statistically significantly higher (*p* = 0.004) than in device immobilized (0.22 ± 0.032, *n* = 16) animals (Fig. 3*G*). The ratio of moving to stationary mitochondria continued to remain high (0.42 ± 0.039, *n* = 14) in levamisole anesthetized animals mechanically trapped by a PDMS membrane (Fig. 3*G*). Similar effects by anesthetics on neuronal transport flux of vesicular cargo have been reported (Mondal et al., 2011).

Using fast time-lapse imaging of the individual TRNs, at a speed of 3 or 4 fps for a total of 1000 frames (time ≥ 150 s), we analyzed turnover rates of moving mitochondria across photobleached stationary mitochondria in the middle of the neuron. The stationary mitochondria drop fluorescence intensity by 96.5 ± 0.89% (from *n* = 20 bleaching events) within 0.33 s, the interval between successive frames at our acquisition rate of 3 fps (Extended Data Fig. 3-1*A–C*). Upon photobleaching a stationary mitochondrion, the motion of a small and faint moving mitochondrion can be easily tracked across the site of the photobleached stationary mitochondrion. In a simple cross event, a small mitochondrion moves across the photobleached mitochondrion without contributing fluorescence to the stationary mitochondrion (Fig. 3*H*; Extended Data Fig. 3-2*D*; Movie 1). While moving mitochondria are identified as contributing to a photobleached stationary mitochondrion by either pausing and/or splitting a portion of their fluorescence leading to fluorescence recovery at the site of the large photobleached stationary mitochondrion (Extended Data Fig. 3-2*E–G*; Movies 2, 3). Sometimes paused mitochondria filled the entire region of the stationary mitochondria area before photobleaching and sometimes they merely pause and then continue to move. Both anterograde and retrograde moving mitochondria were found to contribute to the turnover of stationary mitochondria. On average, more events were observed in anesthetized animals compared with device-immobilized animals across any given bleached mitochondria, likely because of the overall increase in flux in levamisole anesthetized animals (Fig. 3*I*,*J*). Both the device and levamisole immobilized animals show a higher number of anterograde movements across stationary bleached mitochondria as compared with retrograde movements, similar to that observed in movies without bleaching (Fig. 3*E*,*F*; Table 2; Extended Data Fig. 3-2*H*). We also measured total flux as well as a proportion of events in both the initial 1/3 (Init) and the middle (Mid) of the neuron (Fig. 3*K*,*L*). We see the proportion of moving mitochondria is greater in the levamisole immobilized animal in every region of the neuron image (Fig. 3*M*). This might account for the small differences in the number of events where a moving mitochondrion interacts with the stationary bleached mitochondria.

We did not observe any stationary mitochondria split into smaller mitochondria in any of our movies during the entire 4.6 h of imaging either in anesthetic or device immobilized animals. The fluorescence envelopes of moving mitochondria were smaller than stationary mitochondria. We sometimes observed tubular-shaped moving mitochondria in the first 60 μm of the neuronal process proximal to the cell body. Anesthetic immobilization effects perhaps account for the increase in mitochondrial movement which in turn may lead to a higher turnover of mitochondria along the neuronal process.

### Mitochondria are added along the entire *C. elegans* TRN neuronal process

Mitochondria are added to a growing neuron after synapse formation to maintain a constant mitochondrial density (Morris and Hollenbeck, 1993). For example, the number of mitochondria and total process length respectively increased from 7.8 ± 0.41 and 125 ± 6.07 μm in L2 larvae (*n* = 15 animals) to 20.1 ± 1.44 and 335 ± 15.89 μm in L4 larvae (*n* = 10 animals, *p* < 0.01) in the microfluidic device. The neuronal process grows at the rate of ∼10 μm/h and the mitochondria number increases ∼0.6 mitochondria/h, numbers very similar to those seen in animals grown on NGM plates (see above). Although the population statistics indicate average growth rates, it does not indicate the site where new mitochondria are added.

**Figure 4.**
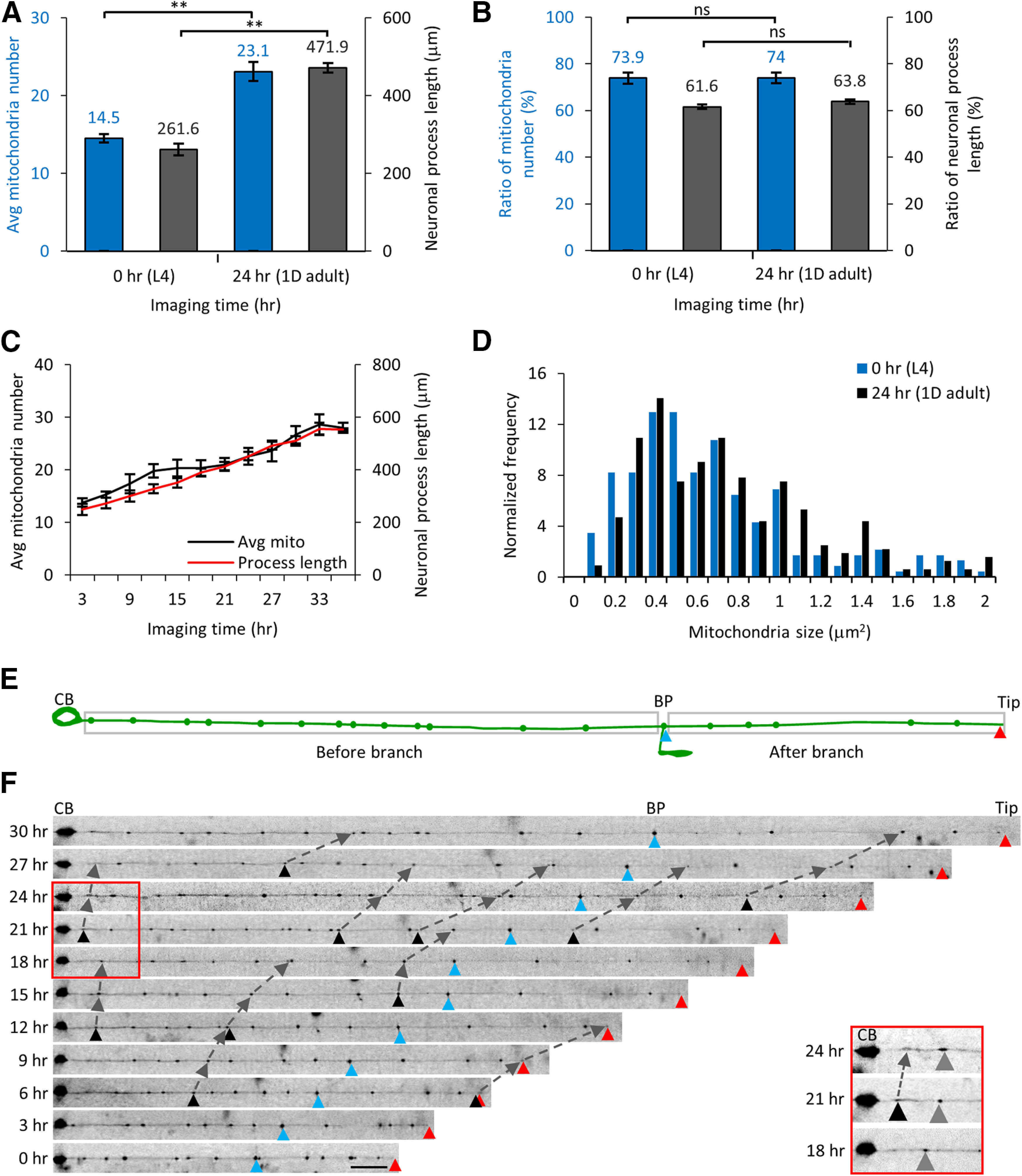
Total number of mitochondria increases linearly in TRNs in animals growing inside microfluidic devices. ***A***, The average number of mitochondria and the neuronal process length increases starting at 0 h (L4) to 24 h (1D adult) of imaging time inside the microfluidic device. ***B***, The ratio of the mitochondria number and the process length was unchanged over this 24 h imaging time period. The ratio is calculated for the values from the cell body (CB) to the BP and from the BP to the neuronal tip. Statistical significance was evaluated by paired sample *t* test; *p < 0.05, **p < 0.005, and ns, *p* > 0.05 when comparing between the 0- and 24-h imaging time point data. ***C***, Time course of the mitochondrial number and neuronal process length from eight animals imaged every 3 h in microfluidic devices beginning at L4 stage. Data represented as mean ± SEM (*n* = 8 animals). ***D***, The relative frequency distribution of all mitochondria sizes at 0- and 24-h imaging time points (*n* = 8 animals). Statistical significance was evaluated by paired sample *t* test (*p* = 0.92). ***E***, A schematic of the TRN shows the neuronal process with the CB, the neuronal process end (Tip, red triangle), and the BP (blue triangle) as fiduciary markers. The BP is used to estimate the total mitochondria number and neuronal process length between the CB and the BP and between the BP and the end of the neuronal process. ***F***, The same animal was trapped inside the microfluidic device every 3 h and mitochondria imaged at high-resolution from the same TRN. Fluorescence images were straightened and raw images are displayed. The fiduciary markers of the CB, BP, and neuronal process end are used as references. New mitochondria added are labeled with black triangles and followed in two successive imaging time points and marked using dashed lines. Scale bar: 20 μm. The red box and the inset show a section of the neuronal process at 18-, 21-, and 24-h time points after start of imaging. A new mitochondrion (black triangle) is added in between the CB and a stationary mitochondrion (gray triangle). The distance between the CB and the stationary mitochondria increases with time because of the growth of the neuronal process. Extended Data Figures 4-1, 4-2, 4-3 support this figure.

To identify the location where the new mitochondria are added as the animal is growing, we imaged the same PLM neuron every 3 h for ∼36 h by repeatedly trapping the animal in our microfluidic device. The images were used to reconstruct the neuron and the location of each mitochondrion was plotted for every imaged time point. The total mitochondria number increased linearly with imaging time. Both, total mitochondria number and neuronal process length at 24 h were significantly higher than at the initial time point (Fig. 4*A*; Table 1). We chose the cell body, branch point (BP), and the neuronal process tip as the fiduciary markers to examine whether there was any bias in the growth of the neuronal process as new mitochondria are added along the neuronal process (Fig. 4*E*). The ratio of the neuronal process lengths between the cell body to the BP and the BP to the neuronal process tip remains constant as the neuron and the animal grows suggesting proportional growth along the neuronal process (Fig. 4*B*,*C*). Over 36 h of imaging, the average total neuronal process growth rate was calculated to be ∼9 μm/h (*n* = 8 animals). We calculated the ratio of the mitochondria number present along the neuronal process between the cell body and the BP to the mitochondria number between the BP and the neuronal tip. The ratio of the mitochondria number remains constant over a 24 h period (Fig. 4*B*), which suggests that new mitochondria are likely added uniformly along the process length.

10.1523/ENEURO.0360-20.2021.f4-1 Extended Data Figure 4-1 Time-lapse images of the neuron from an individual animal. ***A***, The images of the entire neuronal process are shown at 24- and 27-h time points after start of imaging. Two images are aligned with respect to the cell body (CB) and the two additional reference points- the BP (blue triangle) and neuronal end (Tip; red triangle) are marked on the images. The CB, BP, Tip are connected with gray dotted arrows. The trace below the images represents the neuronal processes for both time points with the mitochondria indicated as green dots. The position of fiduciary markers (CB, BP, and neuronal Tip) and three mitochondria are indicated at time *t* =24 h (L_1_, L_2_, and L_3_) and *t* =27 h (L_1_^’^, L_2_^’^, and L_3_^’^) and connected using gray dotted arrows. The ratio of the two distance between given mitochondria from the CB at 24 and 27 h corresponds to the observed 10% (46.6 μm) increase in total neuronal process growth (ΔL = 46.6 μm) in this 3-h period over the initial process length of L_0_ = 443.8 μm at *t* = 24 h. ***B***, Alignment of the neuronal process image of Figure 4*F* for all 11 time points at the tip. ***C***, A representative bubble plot of all 11 time points with the diameter of the bubble proportionate to the total area of each mitochondrion. The highlighted region shows the addition of a new smaller mitochondrion (black arrow and black triangles) between two identified adjacent mitochondria. The small mitochondrion added on 18th hour (black arrow) increases in size in successive imaging frames. A small mitochondrion added at the 24th hour (black inverted triangle) did not persist over 6 h and was not considered as a mitochondrion addition event. Download Figure 4-1, TIF file.

10.1523/ENEURO.0360-20.2021.f4-2 Extended Data Figure 4-2 Mitochondria addition events and intermitochondrial distances measured along the neuronal processes. ***A***, The normalized number of mitochondria added along each segment of the neuron where each segment corresponds to an equal third of the neuronal process. The total number of mitochondria addition events are 85 (9, 10, 10, 10, 11, 15, 14, and 6 for each of the eight animals imaged). Data represented as mean ± SEM. Data were compared using one-way ANOVA with *post hoc* Tukey correction for multiple comparisons (ns indicates nonsignificant for *p* > 0.05). ***B***, Normalized number of intermitochondrial distances in L4 stage animals (*n* = 8 animals) immobilized in the microfluidic chip. The schematic of the mechanosensory neuron with cell body (CB), BP, and neuronal end (Tip). The inset shows a pair of mitochondria (M_1_ and M_2_) with an intermitochondrial distance L. Download Figure 4-2, TIF file.

10.1523/ENEURO.0360-20.2021.f4-3 Extended Data Figure 4-3 Schematic representation of mitochondria addition events between preexisting adjacent mitochondria in a growing neuronal process and the corresponding changes in intermitochondrial intervals after mitochondria additions. No new mitochondria added (***A***), new mitochondria added near the cell body (***B***), new mitochondria added at the end of the neuron (***C***), and new mitochondria added uniformly along the neuronal process (***D***). The addition of a new mitochondrion occurs when the intermitochondrial distance between adjacent mitochondria increases beyond a threshold. Three different time points are denoted that are sequentially (*t* = 0, *t* = T_1_, and *t* = T_2_). The red arrow points to newly added mitochondria. The dashed arrows are added to highlight the position of each stationary mitochondrion in successive time points. The panel on the right is a schematic representation of the expected change in intermitochondrial distances for each possible addition paradigm. Download Figure 4-3, TIF file.

To capture the position and size of every mitochondrion, we reconstructed the entire neuronal process from a series of fluorescence images at multiple time points and analyzed various parameters such as mitochondrial number, size, and the intermitochondrial intervals between each adjacent pair of mitochondria. ∼20% of mitochondria move in any given frame in the TRNs with an average velocity of ∼200 nm/s (Fatouros et al., 2012). Moving mitochondria are less than ∼1 μm in diameter. Stationary mitochondria are bigger in size but drift gradually over time as the animal grows and the neuronal process commensurately proportionately elongates. Small moving mitochondria have low fluorescence intensity and appear randomly and intermittently along the TRN processes in our long-term images because of large time intervals between successive frames. The size of the stationary mitochondria in the neuronal process remains unchanged over longer time scales, i.e., 24 h (Fig. 4*D*). We consider a pair of mitochondria to be identical and stationary if the position of each mitochondrion from the cell body, BP, and tip of the neuron changes proportionately to the average growth rate of the neuron for that animal (typically ∼27 μm per 3 h). The neuronal processes of each individual TRN at multiple time points are aligned with each other from the cell body where the cell body, the BP, and the neuronal process tip act as reference points (Fig. 4*F*). With the growth of the neuronal process, the fiduciary markers (BP and tip) and all the mitochondria move further away from the cell body with time (Extended Data Fig. 4-1*A*). For every mitochondrion, the ratio of the new position to the old position was 1.1 ($L_{i}^{\prime }/L_{i}$ where, $L_{i}^{\prime }$ and $L_{i}$ are the new and old position of the $i^{th}$ mitochondria with respect to the cell body), correlating with the 10% increase in the total neuronal process length over 3 h. The uniform increase of the neuronal processes and the gradual shift in the stationary mitochondria positions are independent of the alignment of the neuronal process with respect to the cell body or the tip of the neuron (Extended Data Fig. 4-1*B*).

To identify a new mitochondrion addition, we identify a mitochondrion between a pair of preexisting stationary mitochondria with higher fluorescence than the background intensity from the neuronal process. The new mitochondrion must stay in the same location for at least two successive frames (i.e., >6 h). The addition of a new mitochondrion was identified using manual inspection of the relative mitochondrial position and geometrical parameters (size and integrated intensity) of the new mitochondrion after aligning the axons with respect to the fiduciary markers (Extended Data Fig. 4-1).

The number of mitochondria addition events was calculated for every equal third of the neuronal process, i.e., initial, middle, and end. The number of events in each third of the neuron was similar 33.3 ± 3.24% (*n* = 29), 37.5 ± 2.61% (*n* = 32), and 29.1 ± 3.31% (*n* = 24) with *p *≥* *0.07 (Extended Data Fig. 4-2*A*; Table 2). We see that each new mitochondrion added has an approximate size of 0.47 ± 0.07 μm^2^ (mean ± SEM) when first identified and grew to 0.68 ± 0.11 μm^2^ (*n* = 4 animals and *n* = 24 new mitochondria addition events) after 3 h. The gain in mitochondrial size can come from contributions from either the moving mitochondria or in situ biogenesis. Moving mitochondria along the neuronal processes are likely to initiate a new location and subsequently gain mitochondrial material by fusion-fission of moving mitochondria to form a larger stationary mitochondrion over time in the neuronal process. Using a long-term imaging platform, we were able to identify the addition of new mitochondria in an identified neuronal process.

### Increased intermitochondrial distance leads to the addition of a new mitochondrion in *C. elegans* TRNs

The growth in the neuronal process increases the intermitochondrial distance between each adjacent pair of mitochondria. This growth of the neuron occurs along with new mitochondria addition such that density is maintained. To characterize changes in mitochondria density and identify the site of the new mitochondrion addition, we calculated the intermitochondrial distances between each adjacent pair of mitochondria (center-to-center distance along the neuronal process length) in all eight L4 larval animals (Extended Data Fig. 4-2*B*). The average distance between adjacent mitochondria was 16.7 ± 0.96 μm (mean ± SEM). To determine whether the distribution of mitochondria along the neuronal process was uniform, random, or clustered, we divided the neuronal processes from all eight animals into 20 μm bins and counted the number of mitochondria per bin. The average number of mitochondria per bin = 1.0 with a variance of 1.16 ± 0.59 (mean ± variance, *n* = 101 mitochondria), df = 4, χ^2^ = 10.02, and a confidence level $P_{\left(10.02,4\right)}=0.04$, suggesting a uniform distribution. Mitochondria can be added at different regions of the neuron altering intermitochondrial distances because of addition of new adjacent mitochondrial pairs (Extended Data Fig. 4-3). (1) Mitochondria can be added near the cell body; (2) at the end of the neuron; or (3) along the neuronal process when the intermitochondrial distance between adjacent mitochondria increases beyond a threshold. In either possibility (1) or (2) the intermitochondrial distance histograms will show two peaks, one at shorter and another at longer intermitochondrial distance intervals corresponding to additions in one region of the neuron and with increasing distance between other adjacent mitochondria pairs because of axon growth (Extended Data Fig. 4-3*B*,*C*). In the third possibility, as the mitochondria move apart because of increasing neuronal process length, the entire intermitochondrial distance distribution will shift to higher values (resulting in a low number of events for the short intermitochondrial distance values). In this case, a new mitochondrion will be added in between the old adjacent mitochondria pair and reducing the likelihood of longer intermitochondrial distance values. Hence, we expect to observe a small reduction in shorter intermitochondrial distances but a relatively unchanged longer intermitochondrial distance (Extended Data Fig. 4-3*D*).

**Figure 5.**
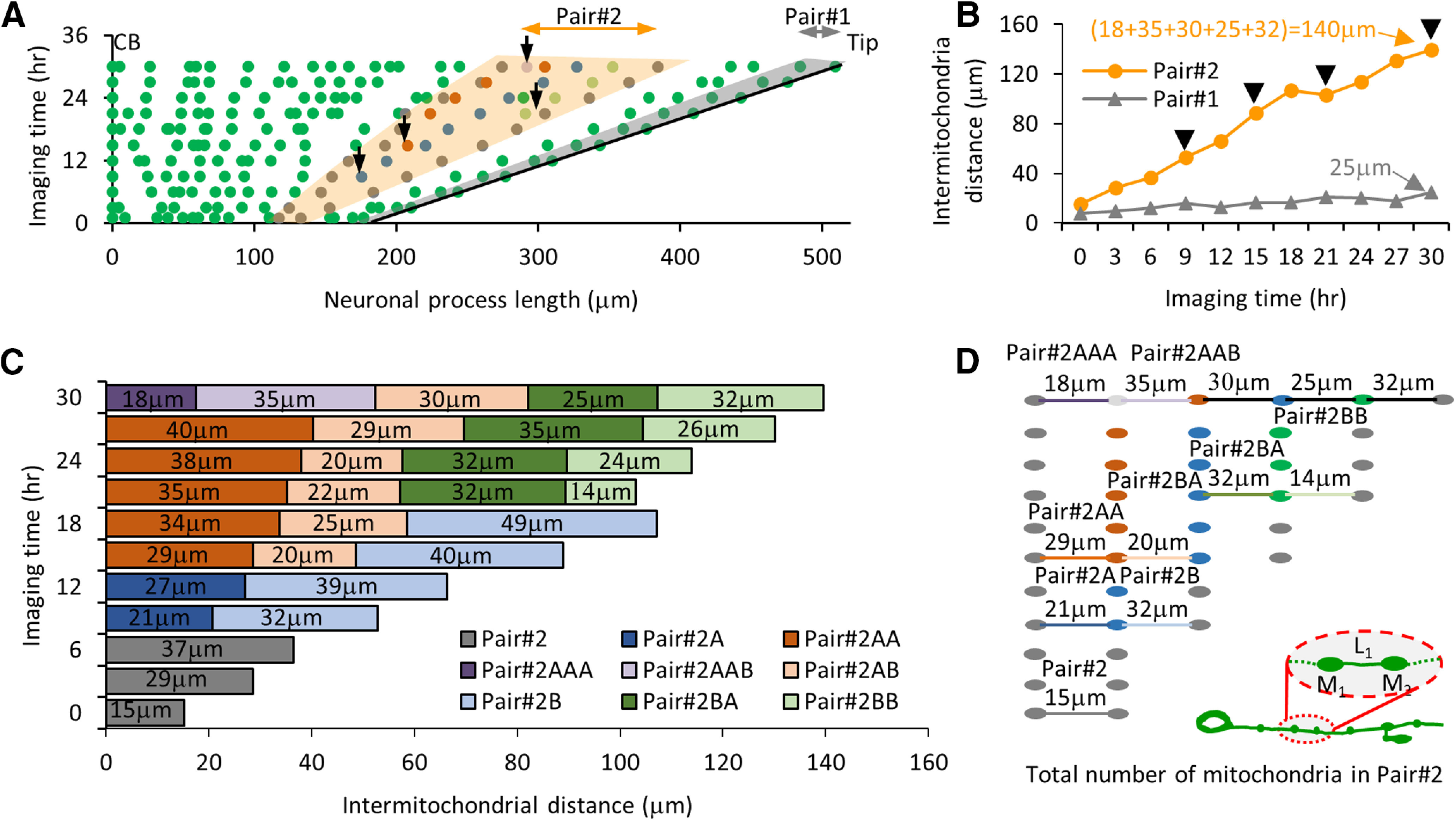
Addition of new mitochondria during long-time imaging of the animal grown in the microfluidic device. ***A***, Map of mitochondrial position from an individual *C. elegans* PLM neuron 36 h after hatching. Solid lines represent all two locations CB and Tip of the neuronal process. The shaded area in gray (pair #1) is a region in the neuronal process where no mitochondrion is added between adjacent pairs in 30 h of imaging. The shaded area in orange (pair #2) represents regions of the same neuronal process where four new mitochondria are identified between the adjacent pairs at different imaging times. The black arrows represent locations of all four new mitochondria. ***B***, Intermitochondrial intervals at different imaging times of two different pairs of stationary mitochondria (pairs #1 and #2) from an individual TRN process. The arrows indicate the time points when a new mitochondrion was added between the two adjacent mitochondria along the TRN process. The values indicate the total distance between parent adjacent pairs at the 30th hour of imaging, on adding all the intermediate intermitochondrial intervals formed because of new mitochondria additions. ***C***, The bar represents the intermitochondrial distances for pair #2 measured from time-lapse imaging. Upon addition of a new mitochondrion, each pair of mitochondria is labeled as new and represented by two new colors (light and dark). The numbers on each bar indicate the intermitochondrial distances for every mitochondria pair. The shorter intermitochondrial distances in a few time points arise because of local compression/expansion of the neuronal process and are considered to be artifacts of imaging because of animal posture. ***D***, Schematic of the mitochondria lineage in pair #2. The gray dots on the right represents the original adjacent mitochondria pair (not to the scale), while the new color dot indicates the location of the new mitochondrion added. Whenever a new mitochondrion is added, the intermitochondrial distances between new adjacent pairs are indicated by a line with a color similar to the color of the bars. The inset shows the schematic of a growing PLM neuron during the development of *C. elegans*. The cell body is on left and the neuronal process end is on right. The inset also shows two mitochondria (M_1_ and M_2_) separated by length L_1_.

**Figure 6.**
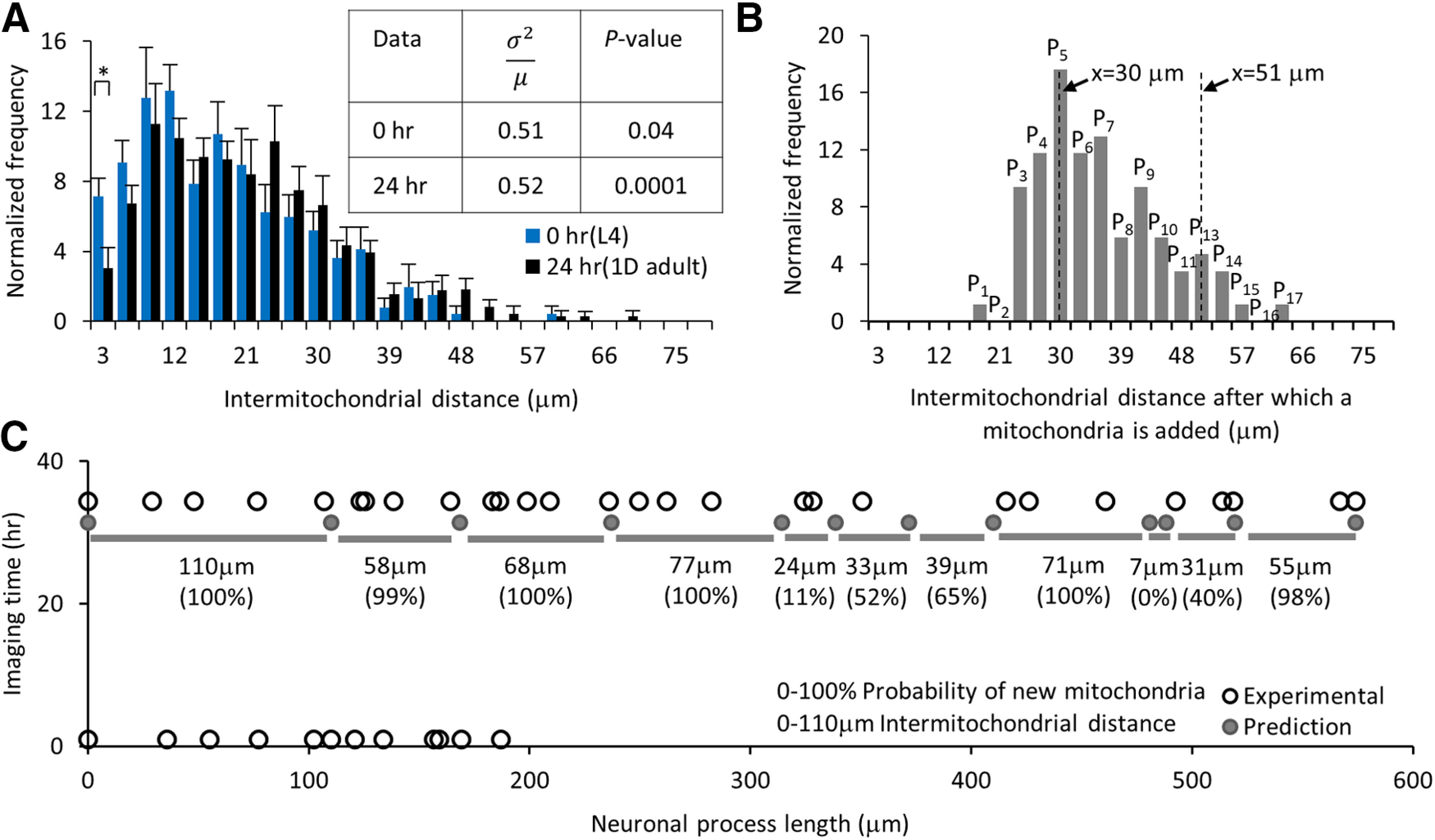
Experimental probability distribution can predict the addition of new mitochondria in a growing neuronal process. ***A***, Normalized number of intermitochondrial distances at 0 h (L4) and 24 h (1D adult) after the start of imaging. Statistical significance was evaluated by two-sample *t* test; *p < 0.05 for the first two bars at bins 3 and 6 μm. The table represents the index of dispersion $\left(\frac{\sigma^{2}}{\mu }\right)$ and *p* value obtained from the goodness of fit estimated using the χ^2^ analysis against a Poisson distribution for data from 0- and 24-h imaging time points (*n* = 8 animals each). ***B***, Normalized number of mitochondria addition is calculated from experimental long-term images of individual TRNs (*n* = 8 animals started at the L4 stage, 85 new mitochondria additions). The probability of new mitochondria added for intermitochondrial distance x = L is shown as P_L_ and can be estimated by summing ($\overset{L}{\underset{i=1}{\sum }}P_{i}$, where i≤L). ***C***, The predicted (dark circle) and experimental (open circle) location of mitochondria at 0- and 33-h images of an individual animal. All the 12 positions at *t* = 33 h are predicted using the locations measured at *t* = 0 h and total neuronal process elongation values. The intermitochondrial distances (gray bars with the values) are used to predict the addition of new mitochondria between each pair at 33 h. The probability values for finding new mitochondria within the predicted pairs are represented in percentages. Extended Data Figure 6-1 supports this figure.

Our data suggest that new mitochondrion addition occurs when the intermitochondrial distance between adjacent mitochondria increases beyond a threshold (orange; Fig. 5*A–D*). An adjacent pair of mitochondria moving apart by 25 μm is still below the threshold and does not dock a new mitochondrion in between them (gray; Fig. 5*A*,*B*). To study mitochondrial addition in a growing neuronal process, we compared the intermitochondrial distances obtained from images at 0 h and 24 h of eight different animals at an early L4 and the 1D adult stages, respectively (Fig. 6*A*). After 24 h, the population average of the intermitochondrial distances between adjacent mitochondria increased from 17 ± 0.7 to 19 ± 0.6 μm (mean ± SEM, *n* = 8 animals). To determine whether mitochondria addition on the neuronal process, continues to maintain a uniform distribution, we performed a χ^2^ analysis with all eight animals at the 24-h time point. We calculated the average number of mitochondria (*n* = 197) per bin = 1.0 at 1.02 ± 0.53 (mean ± variance), df = 4, $\chi^{2}=23.55$, and the confidence level $P_{\left(23.55,4\right)}=0.0001$ suggesting a uniform distribution. This analysis demonstrates that the neuronal processes continue to maintain a uniform distribution of mitochondria, even after multiple additions of new mitochondria. The addition of new mitochondria over 24 h causes a more skewed distribution of intermitochondrial distances as compared with the 0-h time point (skewness and kurtosis measures increased from 0.81 and 0.34 at 0 h to 0.89 and 0.80 at 24 h, respectively). The larger values for the skewness and kurtosis measured at 24 h indicate a strong right-tailed distribution, indicative of non-random mitochondrial positions as the neuronal process grows. Over the same 24 h period, the average values in the first two bins (% of adjacent mitochondria separated by 3 and 6 μm) reduced from 7 ± 1.03% and 9 ± 1.27% to 3 ± 1.17% and 7 ± 1.01% (*p* = 0.02 and *p* = 0.17), respectively (Table 1). No other bin in the normalized intermitochondrial interval histogram was significantly different at the end of this imaging period. Although, lengthening of the neuronal process results in mitochondria moving apart (as indicated by the significant reduction in the shorter intermitochondrial intervals), the addition of new mitochondria ensures an insignificant increase in the larger intermitochondrial distance values. Although mitochondria undergo dynamic membrane fission/fusion and active transport (Sheng, 2014), their addition and positioning along the TRNs were surprisingly found to be regulated as reflected by the highly skewed distribution of the intermitochondrial distance values.

10.1523/ENEURO.0360-20.2021.f6-1 Extended Data Figure 6-1 Intermitochondrial distance statistics when new mitochondria are added between a pair of adjacent mitochondria. ***A***, Schematic of a mechanosensory neuron with the inset highlighting an adjacent mitochondrial pair along the neuronal process. ***B***, Schematic of the neuronal process that shows the increase in the intermitochondrial distance between the pair of mitochondria at three different time points (*t*_0_, *t*_0_ + 3 h, and *t*_0_ + 6 h). A new mitochondrion is added at *t*_0_ + 6 h. The intermitochondrial distance is measured between the old (pair #1) and new adjacent pairs (pairs #1A and #1B) of mitochondria at time points before and after the addition of the new mitochondrion, respectively. ***C***, Box and whisker plot of all the intermitochondrial distances from time-lapse imaging of the neuronal processes of animals (*n* = 8) grown inside the microfluidic chip and imaged for 36 h. The box represents 25th and 75th percentiles, whiskers represent outliers, and the center small box represents the mean (*n* = 85 mitochondrial pairs). ***D***, The bar graph of intermitochondrial distances between new pairs of adjacent mitochondria after the addition of a new mitochondrion between a pair of old adjacent mitochondria (*n* = 85 new pairs). ***E***, The intermitochondrial distance for all 85 new pairs (pairs #1A and #1B) are plotted as a function of the normalized neuronal process location, where an addition event occurred. Download Figure 6-1, TIF file.

Adjacent intermitochondrial separation initiates the docking of a new mitochondrion forming two new adjacent independent pairs of mitochondria. One pair (pair #1A) is with the old mitochondria close to the cell body and second pair (pair #1B) is with the old mitochondria far from the cell body (Extended Data Fig. 6-1*A*,*B*). The intermitochondrial distances between the old mitochondrial pair at the imaging time before a new mitochondrion is added show a distribution with an average interval of 34 ± 1.0 μm (mean ± SEM, *n* = 85 mitochondria addition events; Fig. 6*B*; Extended Data Fig. 6-1*C*). We identified all new mitochondria pairs to estimate the average distance for pair #1A and pair #1B as 17.1 ± 0.8 and 21 ± 1.2 μm (mean ± SEM), respectively for all 85 events (Extended Data Fig. 6-1*D*). The new mitochondria addition events are uniformly distributed along the neuron (Extended Data Fig. 6-1*E*). Consistent with the third possibility (Extended Data Fig. 4-3*D*), we observed 85 events where two adjacent mitochondria had an intermitochondrial interval >24 μm when a new mitochondrion position appeared and remained for six or more hours. The addition of new mitochondria occurs uniformly throughout the neuron and maintains a similar average intermitochondrial distance with its adjacent mitochondria over a day of long-term imaging.

### Mitochondria positions can be estimated from the experimental probability distribution function

Using all the individual events where a new mitochondrion was added, we calculated the experimental probability distribution of new addition events and predicted possible locations for the addition of new mitochondria using this probability distribution. The normalized plot of intermitochondrial distance (Fig. 6*B*) gives us the experimental probability distribution with a sum of all the values ($\sum^{}P$) = 100%. For any mitochondrial pair, we calculate the intermitochondrial distance (x=L) and add normalized numbers of mitochondrial addition for all the intermitochondrial distances lower than the current value to assign the probability ($P_{L}$) for the addition of a new mitochondrion between a given adjacent pair ($P_{L}=\overset{L}{\underset{i=1}{\sum }}P_{i}$, where i≤L). To test our experimental probability distribution values, we imaged a neuronal process length (L_0_ = 186.7 μm) with total mitochondria (N_0_ = 12) at time *t* = 0 h and predict that the hypothetical neuronal process grows to a length (L_33_ = 573.7 μm) at time *t* = 33 h after the start of imaging. This gave us 12 images that were collected every 3 h (Fig. 6*C*). For the “predicted” neuron, we calculate that the neuronal process elongates steadily with an experimental rate of 11 μm/h, similar to the mean neuronal process elongation rate of ∼9 μm/h. Using the above parameters, we estimate that the neuronal process (with an average intramitochondrial distance of 17 ± 2.8 μm at *t* = 0 h) is predicted to grow ∼11 μm/h in 33 h leading to an average intermitochondrial interval of 52 ± 8.7 μm, without the addition of new mitochondria (Fig. 6*C*). When the separation between adjacent mitochondrial pairs increases beyond 24 μm, we predict the addition of a new mitochondrion (Fig. 6*B*). This prediction is supported by our experimental observations (Fig. 6*C*). The addition of a new mitochondrion between two stationary mitochondria, as the neuronal process expands with the growth of an animal, is controlled by the rate of neuronal elongation, a gradual increase in the intermitochondrial distances beyond the 24 μm threshold, and predicted by the experimental probability distribution.

This technology allows us, for the first time, to track individual neurons *in vivo* to assess dynamics that occur over slow timescales such as intracellular organelle positioning across development. Using our long-term imaging device, we were able to extract the experimental probability distribution for organelle addition along the axon and use the values to predict the location of a new mitochondrion addition between a pair of adjacent preexisting mitochondria in a growing neuron. Multiple factors could determine the addition of new mitochondria such as the ratio of molecular motors (Sure et al., 2018; Chen et al., 2021), local cytoskeleton (Sood et al., 2018), local calcium concentration (Wang and Schwarz, 2009b), new docking sites (Cai and Sheng, 2009), and/or reactive oxygen species (Debattisti et al., 2017; Liao et al., 2017).

### Our long-term device can be used to study synaptic development in an individual animal

**Figure 7.**
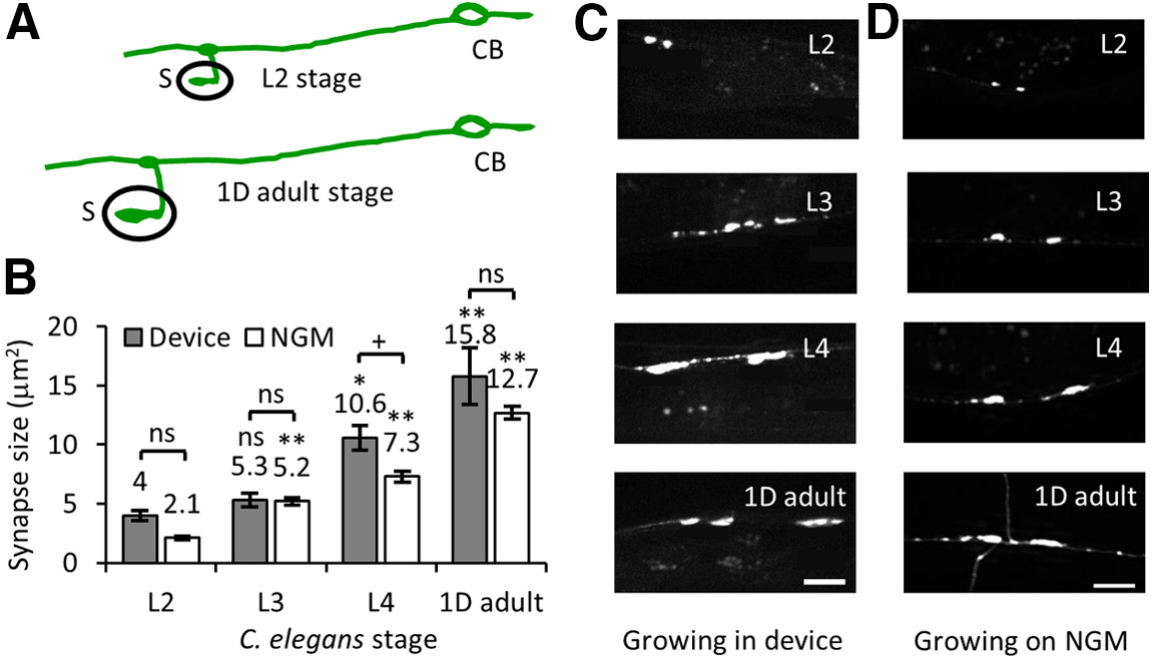
High-resolution imaging of GFP::RAB-3 in a developing *C. elegans* inside the microfluidic device. ***A***, Schematic of PLM neuron with its cell body (CB), its neuronal process, and its ventral synapse (S) at L2 larva and 1D adult stages. ***B***, The plot of synapse sizes at different developmental stages of *C. elegans* growing inside a microfluidic device and on NGM plates. Data represented as mean ± SEM. Statistical significance was evaluated by one-way ANOVA with Bonferroni *post hoc* comparisons; *p < 0.05 and **p < 0.005 when compared with data from L2 stage; +*p* < 0.05 and ++*p* < 0.005, ns, *p* < 0.05 when compared between animals grown in the device and on NGM solid media; nonsignificant comparisons are not indicated. The number of synapses analyzed from animals grown on NGM solid media is *n* ≥ 37 and in devices are *n* = 18 (L2), 16 (L3), 14 (L4), and 8 (1D adult). ***C***, ***D***, Images of PLM synapses at L2, L3, L4, and 1D adult stages of development grown in the microfluidic device (***C***) and on NGM solid media plates and imaged on agar pads (***D***). Scale bar: 10 μm.

We wanted to test whether our device could be used to image other developmental processes to examine its general utility for other types of biological processes. To perform high-resolution time-lapse imaging of subcellular organelle accumulation events in *C. elegans* across developmental stages, we captured fluorescence images of GFP::RAB-3 accumulation at the ventral PLM synapses from the same individual *jsIs821* animals (Nonet, 1999; Mahoney et al., 2006; Kumar et al., 2010). We immobilized each animal using our imaging platform under 14 psi pressure inside the flow channel. Slow progressive immobilization provides a suitable lateral orientation of *C. elegans* and the best view of the PLM synapses (Fig. 7). We immobilized the same animal in a lateral orientation to image both PLM synapses at L2 (16 h), L3 (22 h), L4 (38 h), and 1D adult (68 h) stages from the time the eggs hatched. High-resolution time-lapse images were analyzed for synapse sizes to estimate synapse growth at different developmental stages. The amount of GFP::RAB-3 accumulation shows a gradual increase similar to the prior average signal measured from multiple populations of *C. elegans* animals immobilized inside the microfluidic device at different developmental stages (Mondal et al., 2011). This increase is thought to arise from the gradual increase in the net anterograde flux of SVs (Mondal et al., 2011). Our imaging platform captured a robust increase in the accumulation of GFP::RAB-3 at the synapses of the same animal over multiple developmental stages.

## Discussion

*C. elegans* TRNs grow from ∼100 μm at the L1 larval stage to 500 μm in 1D adult stage. During this growth, ∼20 new mitochondria are added to maintain a constant mitochondrial density of approximately five mitochondria/100 μm along the neuronal process (Fig. 1*D*,*E*). The density measured in adult animals grown in our microfluidic device is very similar to that previously reported in young adult animals (Morsci et al., 2016; Sure et al., 2018). Conventional anesthetic assays can have detrimental effects on animal physiology, i.e., it can alter muscle tone consequently altering body length, and cannot easily be used for long-term imaging of the same animal. Anesthetic-free immobilization using a microfluidic device shows robust vesicle transport (Mondal et al., 2011), fast axonal regeneration rates (Guo et al., 2008), and quick behavioral recovery (Dong et al., 2018; Manjarrez and Mailler, 2020). In agreement with our previous vesicle transport studies (Mondal et al., 2011), we found robust mitochondrial transport in the device immobilized animals. To follow slow mitochondria addition during neuronal process growth, we developed a microfluidic device that can be used to grow *C. elegans* throughout its development without significant adverse effects on its growth and health (Fig. 2*A–F*). Its easy operation enables continuous imaging of the same animal inside the microfluidic environment to quantify *in vivo* subcellular process in the neuron throughout development. Mitochondria in the TRNs were found to show lower flux in the microfluidic device compared with anesthetic immobilization (Fig. 3*D*). These observations are similar to the lower flux of SVs when immobilized in microfluidic devices compared with anesthetic immobilization (Mondal et al., 2011). Using long-term imaging over a period of 36 h, we identified mitochondria addition and changes in intermitochondrial distances in *C. elegans* TRNs during its elongation after synapse formation (Fig. 4*F*). Using our microfluidic device platform, one can monitor slow processes such as the addition of mitochondria. Slow entry and exit and limited turn-over of mitochondria identified in our imaging platform suggest that the neuron retains positions of old mitochondria and adds mitochondria at new locations between preexisting mitochondria along the growing process during development (Fig. 4*F*).

New mitochondrion addition during growth and axon stretching has been shown to occur between adjacent pairs of stationary mitochondria (Miller and Sheetz, 2004; Lamoureux et al., 2010; Misko et al., 2012; Matsumoto et al., 2020). Our data are consistent with prior observations where we see a uniform distribution of mitochondria, proportionate growth of the neuron, and addition of mitochondria occurring anywhere along the process between two stationary mitochondria (Fig. 5). This mitochondrion addition occurs anywhere along the neuronal process whenever the average separation between two nearest stationary mitochondrial neighbors is at least 24 μm (Fig. 6*B*), suggesting the presence of a signal that detects intermitochondrial distances.

We have identified the experimental probability distribution for the addition of a new mitochondrion in a growing neuronal process *in vivo* over a 24 h period. The distribution for the intermitochondrial distances becomes more skewed and the mean intermitochondrial distance increases with the development of the animal (Fig. 6*A*). Although new mitochondria are added over the 24-h time, the index of dispersion indicates that the distribution remains uniform (Fig. 6*A* table) strengthening the argument that addition is not a random process. As development and growth occur, the number of closely spaced mitochondria is reduced, decreasing the fraction of mitochondria with lower separation between them. During neuronal process elongation, the new mitochondria added are small in size and have low integrated fluorescence intensity, they increase in size and intensity over time (Extended Data Fig. 4-1). There are two possible mechanisms for the addition of a new mitochondrion that remains a subject for further studies: (1) a small mitochondrion enters from the cell body, moves along the neuronal process, identifies a large intermitochondrial separation, and anchors within the “mitochondria-in-demand” region; and (2) a local mitochondrion fission event causes a new small mitochondrion to emerge from a nearby large mitochondrion, moves a shorter distance, and anchors within the mitochondria-in-demand region. However, we have not observed a large stationary mitochondrion show such fission in our imaging. All events appear to be initiated by moving mitochondria undergoing fusion or fission with a preexisting stationary mitochondrion.

Stationary mitochondria when photobleached using a high-power laser, lose nearly 96.5% of the fluorescence intensity instantaneously (in 0.33 s when captured at 3 fps rate; Extended Data Fig. 3-2*B*). We think our bleaching protocol is unlikely to damage mitochondria sufficiently to make them generally permeable to cytoplasmic GFP or GFP coming from another mitochondrion that crosses this bleached stationary mitochondrion. We observe several examples where rapidly moving mitochondria cross photobleached mitochondrion locations without any significant change in fluorescence intensity of the moving or the photobleached mitochondria (Extended Data Fig. 3-2*D–F*). Only a subset of moving mitochondria interacted with a photobleached mitochondrion.

Halting of a mitochondrion at the new mitochondria-in-demand region is likely to be facilitated by several factors such as motors, local calcium levels, local ATP concentrations, cytoskeleton architectures, etc. Thus, the addition of new mitochondria likely arises from the stalling of moving mitochondria at a location, between a pair of previously docked mitochondria, with a probability that increases with the increasing separation between two adjacent stationary mitochondria. The stalled mitochondria appear to enlarge in size potentially through fusion with material from moving mitochondria that go past it. We believe that time-lapse imaging of adjacent mitochondrial pairs >24 μm apart, identified from our long-term time-lapse imaging platform, could shed light on mechanisms of mitochondrial addition. Time-lapse imaging of fission mutants and animals with transport defects can also help delineate the underlying processes and molecular players important in new mitochondria addition events. The long-term mitochondria dynamics studies in *C. elegans* models using a microfluidic platform not only offers a robust assay to study organelle transport *in vivo*, but it provides an opportunity to move beyond simple observations obtained from *in vitro* assays to discover genes that can regulate the positioning of mitochondria in neurons.

Our device can be used at different time intervals. For studies that require more frequent imaging of the same animal, we immobilize the same animal every hour for 12 h (Extended Data Fig. 3-1). A 1 h more frequent immobilization showed greater but statistically insignificant photobleaching when imaged at 12 h time point (Extended Data Fig. 3-1*C*). Intermittent long-term imaging separated by ∼6 h for 52 h shows a steady increase in synaptic accumulation of GFP::RAB-3 when measured from an individual animal grown inside the microfluidic device. The amount of accumulation was slightly higher in device-grown animals as compared with animals grown on NGM plates (Fig. 7; Mondal et al., 2011). This increase compared with animals grown on NGM plates may arise from additional mechanical stimulation provided by the deflected membrane pressing against the animal. Despite these caveats, our microfluidic platform can be used for a variety of studies to assess long-term cellular or subcellular dynamics in *C. elegans*. Similar technologies can be used for other model organisms by modifying the channel geometries (Mondal et al., 2011, 2012).

## Synthesis

Reviewing Editor: Miriam Goodman, Stanford University Decisions are customarily a result of the Reviewing Editor and the peer reviewers coming together and discussing their recommendations until a consensus is reached. When revisions are invited, a fact-based synthesis statement explaining their decision and outlining what is needed to prepare a revision will be listed below. The following reviewer(s) agreed to reveal their identity: Kyle Miller, Linda Dansereau.

The authors have demonstrated the feasibility of long-term imaging of organelles in animals trapped in a microfluidic device compared to anaesthetised animals. Using the device they have shown that new mitochondria in touch receptor neurons are added between two existing mitochondria as the neuron grows rather than entering from the cell body. They also show that synapses could be studied using this device. The reviewers appreciated both the technical advance and the observations presented, noting that the study is likely to be of interest to neuroscientists with interest in neuronal development, mitochondrial dynamics, and organelle trafficking. In parallel, they also noted opportunities for improvement following a revision. Below, I include the full reports from both reviewers and ask that you focus your efforts on these key elements.

1. Include a broader discussion of prior studies in the introduction and discussion

2. The claim that the mitochondria are not distributed randomly needs to be better supported or revised.

3. Consider adopting eNeuro’s recommendation to use ‘estimation statistics’ to address the reviewers’ comments regarding significance, effect size, and power. https://www.eneuro.org/lookup/doi/10.1523/ENEURO.0259-19.2019

4. Revise the composition to address some awkward phrases that are found throughout this submission (e.g. The opening sentence of the Significance Statement has an error in the agreement of number - “How does new stationary mitochondrion form into a growing neuron?”)

5. For mobile mitochondria, please report flux and velocity.

6. If possible, perform an experiment in which animals immobilized in the chip are exposed to levamisole. This will help to clarify if the reported differences in mitochondria transport arise from a common effect (e.g. mechanical stress induced by immobilization or by levamisole-induced increase in body muscle tone. Levamisole is known to increase body stiffness - Petzold et al, 2013) or are distinct from one another.

7. eNeuro style does not use supplemental figures, so the panels labeled as 2-1, 3-1, 3-2, etc will need to be reformatted. Supplemental panels that are tables (e.g. 3-3) should be reformatted as tables

Rev1

In the paper entitled “Tracking mitochondrial density and positioning along a growing neuronal process in individual C. elegans neuron using a long-term growth and imaging microfluidic device,” the authors pose the question of how organelles are added to growing axons. They then describe the development of a microfluidic device that allows repeated high-resolution visualization of organelles in vivo in C. elegans in single animals over days. Focusing on mitochondria, they report that as axons lengthen in vivo, the distance between pairs of docked mitochondria increases over time, presumably reflecting axonal stretching as the result of body growth. When the average separation between two mitochondria exceeds 24 um, a new mitochondrion is added between them, which occurs along the entire length of the axon. In addition, as mitochondria are actively transported along the axon, changes in fluorescence intensity suggest transient mitochondrial fusion and fission events may be occurring with docked mitochondria. Extending this approach, they demonstrate that the accumulation of synaptic markers can be monitored in individual animals over time. Overall this is a well-written manuscript with high-quality experimental data. I expect that it will be well received by those interested in both neuronal developmental and mitochondrial biology.

The main suggestions in brief are:

1. To include a broader discussion of previous studies, in both the introduction and discussion, to better place this work in context.

2. To add statistical tests to more directly make the case that axonal mitochondria have a non-random distribution.

3. The addition of an experiment where the paralytic agent Lev and immobilization are applied simultaneously, to test if Lev increases mitochondrial transport or immobilization suppresses it.

4. Determine if mitochondrial intensity drops slowly or suddenly during photobleaching.

5. Report mitochondrial flux and velocity.

6. A check to determine if using the segmented line tool in ImageJ is creating an artifact when measuring distance.

7. Do a test for statistical power for figure 4.

Abstract, Significance Statement, and Introduction

There are numerous grammatical mistakes through-out the manuscript. Running the text through Grammarly would help correct many of these. In addition, a careful copyedit would improve readability. To show just one example, the first sentence of the Significance Statement is, “How does new stationary mitochondrion form into a growing neuron?"

My understanding is that the significance statement should describe why the work is important, rather than repeating the findings stated in the abstract.

In the first paragraph of the introduction, it feels like an oversight not to mention that Miller and Sheetz (2004) and Misko et al., (2012) have both reported mitochondria have a uniform distribution along the axons. In addition, Miller and Sheetz (2004) conducted an analysis that suggested the uniform distribution arises because rapidly transported mitochondria tend to dock in the center of pairs of pre-existing docked mitochondria. As those experiments were conducted over the course of about an hour in neurons from different animals, the statement “To investigate where mitochondria are added, one needs to analyze each mitochondrion over days in the same animal” seems incorrect. Placing this work in the context of prior studies in no way undermines its contributions. Instead it shows that the findings reported here are robust and relevant to the development of vertebrate neurons.

More generally, the results in this manuscript are of interest to the problem of how axons lengthen during body growth. The following two reviews address this problem to varying degrees:

Stretch growth of integrated axon tracts: Extremes and exploitations, by (Smith, 2009)

Axonal cytomechanics in neuronal development (Mutalik and Ghose, 2020)

Of these, Mutalik and Ghose is both recent and has a particularly well-referenced section on body growth and axonal elongation. Adding a few sentences in the introduction / discussion about this problem would help illuminate the importance of the experimental work presented here. In this context, Fig. 4F is one of the best illustrations, I’m aware of, that body growth causes axons to stretch and new material is added along the axon. Another paper, that you might consider citing is Lamoureux et al. (2009). It consists of a series of experiments, which suggest new mass and mitochondria are added along the axon to compensate for axonal stretching that occurs during neurite outgrowth.

In the methods, it says animals were immobilized using Levamisole, while in the results it at one point, azide is mentioned, which is correct?

Fig. 2-1 helps in explaining Fig 2A-C. Could it be used in the main figure?

Where it says, “less than 3 pixels in 5 consecutive frames and (2) moving if their displacement is equal to or more than 3 pixels in less than 5 consecutive frames.” What are the distances in microns and the time step between frames? I.e., what is the upper bound on velocity to be considered stationary?

In terms of understanding why mitochondrial transport is higher in Levamisole treated worms, as opposed to worms immobilized in the device without drugs, Levamisole is a nicotinic acetylcholine receptor agonist that causes intense stimulation that leads to paralysis. Thus, I wonder if higher levels of synaptic activity increase ATP demand and thus upregulate mitochondrial transport to supply mitochondria. In light of this, it seems important to combine 3mM Lev and immobilization with the device at the same time. If the number of moving mitochondria were at the same high level as seen with 3mM Lev alone when Lev is added to worms in the device, it would suggest that Lev is increasing mitochondrial transport. In turn, if the level of transport were low, as in the device alone, it would indicate the device is reducing transport. Doing such an experiment would help to determine if the following statement is correct, “The high amount of stress introduced to the animal in an anesthetic assay perhaps accounts for the increase in the mitochondrial activity which may cause a high turnover of mitochondria along the neuron.” To underline the importance of this, on my first read through, my impression was that immobilization might be damaging the animal and reducing transport. After a more careful read, I’m now inclined to think that Lev might induce artificially high levels of mitochondrial transport.

Looking at mitochondrial fusion-fission using photobleaching in Fig. 3-2 is very interesting. A couple of points. When the mitochondria are photobleached, do they slowly decrease in fluorescence, or does the drop in brightness occur suddenly? Likewise, would it be possible to include a systematic analysis? If the later, this suggests a mitochondrial permeability transition has occurred. There are two implications. The first is that the mitochondrion is no longer producing ATP, which might act as a signal for docking. The second is that the sudden drop in fluorescence in Fig. 3-2A, for the rapidly moving mitochondrion, might be occurring because it transiently fuses with the damaged mitochondria. As there are ‘holes’ in the damaged mitochondria, when GFP is being transferred to one mitochondrion to another, the GFP just leaks out into the cytoplasm. I wouldn’t mind a bit more discussion of these results.

Fig. 3F and G, which look at transport, are interesting as well. My take away is that in the immobilized animals, essentially, all of the transport is in the anterograde direction, and the addition of Lev increases transport in both directions. Because mitochondrial transport is central to this manuscript, the inclusion of the values for average flux and instantaneous velocity for the conditions shown in 3D seems warranted; this could be added to table 3-3. In addition, the values for the immobilized condition should be added to the table. If I understand fig 3D correctly, the anterograde flux in the device is 1.6 mito/hr, and the retrograde flux is 1 mito/hr. Collectively, this indicates the net flux is 0.6 mito/h anterograde, which indeed matches up nicely with the observed increase in mitochondrial number discussed in Fig. 4. It would strengthen the manuscript to make this link easier to follow.

With regards to the statement, “The uniform increase in neuronal process elongation (Fig. 4C) is consistent with the earlier studies that show uniform membrane addition in the growing neuron process (Popov et al., 1993, Chen et al., 2014).” Where the plasma membrane is added to growing axons has been somewhat controversial. As a window into this, there is a review from Caceres’ group by Quiroga (2017) that argues new membrane is added at the growth cone. I haven’t been directly involved in this debate, but my take is that is some cases, new membrane is added along the axon. In others, it is added mostly at the growth cone, and in some cases, it may be mainly added to the cell body. I would suggest avoiding this controversy about the site of membrane addition, as it doesn’t seem very relevant to mitochondrial addition. Likewise, while Chen, 2014 is an excellent paper, it isn’t directly relevant to the sub-cellular site of membrane addition. If you are looking for a manuscript that suggests mitochondrial addition occurs along the axon, please take a look at Lamoureux et al., 2009 (Dev. Neu.). Essentially, the entire manuscript is focused on this one point and includes an analysis of mitochondrial addition to growing axons.

To preface, Fig. 4F is beautiful! Nonetheless, I do worry that there might be an artifact caused by an odd bug in ImageJ where the straighten function incorrectly returns the distance between objects when using the segmented line tool to trace objects. In contrast, it correctly returns distance when using the freehand line tool. For example, going from 15 to 18 hr, the branch point does not move while the tip advances. Is this ‘real’ or an artifact caused by a bug in ImageJ? To test, please take the original data in Fig. 4F and trace the axons in ImageJ using both the Freehand line selector and the Segmented Line tool. If the results differ significantly, please consider reanalyzing the data set. In doing so, it is possible that the position of the docked mitochondria will line up more tightly over time. Of note, this problem causes the largest issues when the length of the segments along a line varies significantly. This is to say, if the segments of the same length are used to trace an object, then the results are typically very similar to those seen using the freehand line tool.

In the legend for Figure 4-2, it states, “The p-value {greater than or equal to} 0.7 estimated from Student’s t-test for each pair indicates that the mitochondria are added uniformly along the neuronal process length.” As three segments are being analyzed, a one-way ANOVA with post-hoc Tukey should be conducted. Likewise, when making a case for ‘non-significance,’ the statistical power should be stated. Somewhat confusingly, in the text, a p-value of 0.07 is noted. If p = 0.07 is the correct value, it is improbable that there is the statistical power to claim no difference in the addition between the three segments with a substantial degree of confidence. If there is a misunderstanding here on my part, the text and figure need to be rewritten to make the arguments easier to follow.

With regards to Fig. 4, Fig. 5, and Fig. 6, which address how new mitochondria are adding to growing axons, Miller and Sheetz (2004), Misko et al. (2012), and Matsumoto (2020, unpublished, bioRxiv) have all indicated that mitochondria have a uniform distribution along axons. In these terms, applying the same statistical test used by Miller and Sheetz (2004) and Misko et al. (2012) would provide a more direct argument that mitochondrial distribution is uniform. Additionally, this will allow a more direct comparison of these new data with the older studies. Likewise Fig. 4-3, and Fig. 5 are nicely constructed but are somewhat difficult to follow. While I don’t think they need to be removed or replaced. Adding a more rigorous statistical analysis of mitochondria distribution (i.e., calculation of the index of dispersion paired with a Chi-squared analysis against a Poisson distribution; as used in previous papers) would help strengthen the argument that mitochondria have a non-random organization in axons.

In terms of the Discussion:

Adding a discussion of what happens when immobilization and Lev occur simultaneously will strengthen the first paragraph, which is focused on the development of the device. In particular, these additional data have the potential to strengthen the case that immobilization provides a more physiologically relevant view of mitochondrial transport than when Lev is used to paralyze animals.

The second paragraph of the discussion focuses on the way that mitochondria are added to the axon. Including a statistical analysis of the index of dispersion paired with a Chi-squared analysis would strengthen the case that mitochondrial addition along the axon is not a random process.

A relatively major point in this section of the discussion concerns the pattern by which new mitochondria are added to the axon. To the best of my knowledge, Miller and Sheetz (2004) is the first paper which suggested mitochondrial docking occurs between pairs to previously docked mitochondria. Further, that paper was the first to propose that axonal mitochondrial distribution is uniform, which was later confirmed by Misko et al., (2012). Discussing the results of this current paper in the light of these previous findings would help to strengthen the case that either a protein complex on the mitochondria or along the axon somehow senses ATP levels or the presence of other mitochondria and engages docking to control the distribution of mitochondria. In addition, because those experiments were conducted in vitro using vertebrate neurons, whereas this study was conducted in vivo in invertebrates, a strong case can be made that the findings reported here are robust and relevant to other systems. Given there is another unpublished paper on bioRxiv indicating axonal mitochondrial distribution is uniform, my feeling is that there is growing excitement about this problem. With the excellent genetic tools in C. elegans there is now the opportunity to move beyond simple observations to discover the genes that regulate the positioning of mitochondria in neurons.

As a final comment, reading the abstract, one gets the impression that this primarily a methods paper. Nonetheless, I see several exciting biological discoveries here. In particular, in terms of the relationship between body growth and axonal lengthening and the regulation of mitochondrial addition to the axon. A slight rewrite of the abstract to better convey these exciting results would help ensure that more people read this manuscript.

Rev2

The authors have demonstrated the feasibility of long-term imaging of organelles in animals trapped in a microfluidic device compared to anaesthetised animals. Using the device they have shown that new mitochondria in TRNs are added between two existing mitochondria as the neuron grows rather than entering from the cell body. They also show that synapses could be studied using this device.

Major comments:

1. In the section about “Growth in a microfluidic device does not impede C. elegans development” there are two sentences that make essentially the same reference to Keil et al., 2016 concerning slower growth of C. elegans in liquid culture. The second reference is made without without directly relating to the data presented in the paper. The authors do not make a direct comparison on how the length and width of the animals grown in the microfluidic chamber compare to the reported qualities of animals grown in liquid culture. It would also be interesting to compare synapse development in animals grown in liquid culture with those grown in the device.

2. In Figure 3D the authors use a bar graph to show the number of times mitochondria enter or exit neurons in anaesthetised animals versus device immobilized animals. Visually the differences look like they might be significant but the p-value is stated as either > 0.5 (in text) or {greater than or equal to} 0.055 (in the legend). It is also unclear whether the error bars represent standard error or standard deviation which makes it difficult to assess. Including the individual points in the graph, or use of a box-whisker plot, will more clearly show why the differences may not be significant.

3. It is interesting that while the difference in the number of moving mitochondria is not significant, the difference in average events/hr (as shown in Fig 3F-G) is significant. An additional analysis looking at the ratio of events to the number of moving mitochondria would be useful to show if the proportion of events is the same or different between the two conditions.

Minor comments:

1. In general, the figures are clear and well presented, however there are a few figures and figure legends that could be revised. These include:

a. A missing description of red arrows in the legend of Fig 2E 84 hr timepoint.

b. Changing the order of Device and Lev bars in Fig 3D to match the order in Fig 3F-G.

c. Adding a bleach time marker to the kymographs where photobleaching was used

d. Individually colouring the relevant dots in Fig 5A to match Fig 5D would make the figure easier to interpret. Adjusting the shaded yellow triangle slightly to more accurately include the relevant dots would work as well.
