# Supplementary figures and images for: Tracking Mitochondrial Density and Positioning along a Growing Neuronal Process in Individual C. elegans Neuron Using a Long-Term Growth and Imaging Microfluidic Device
Source: eNeuro. 2021 Jul 2;8(4):ENEURO.0360-20.2021. doi: 10.1523/ENEURO.0360-20.2021 (PMC8260276; doi:10.1523/ENEURO.0360-20.2021)

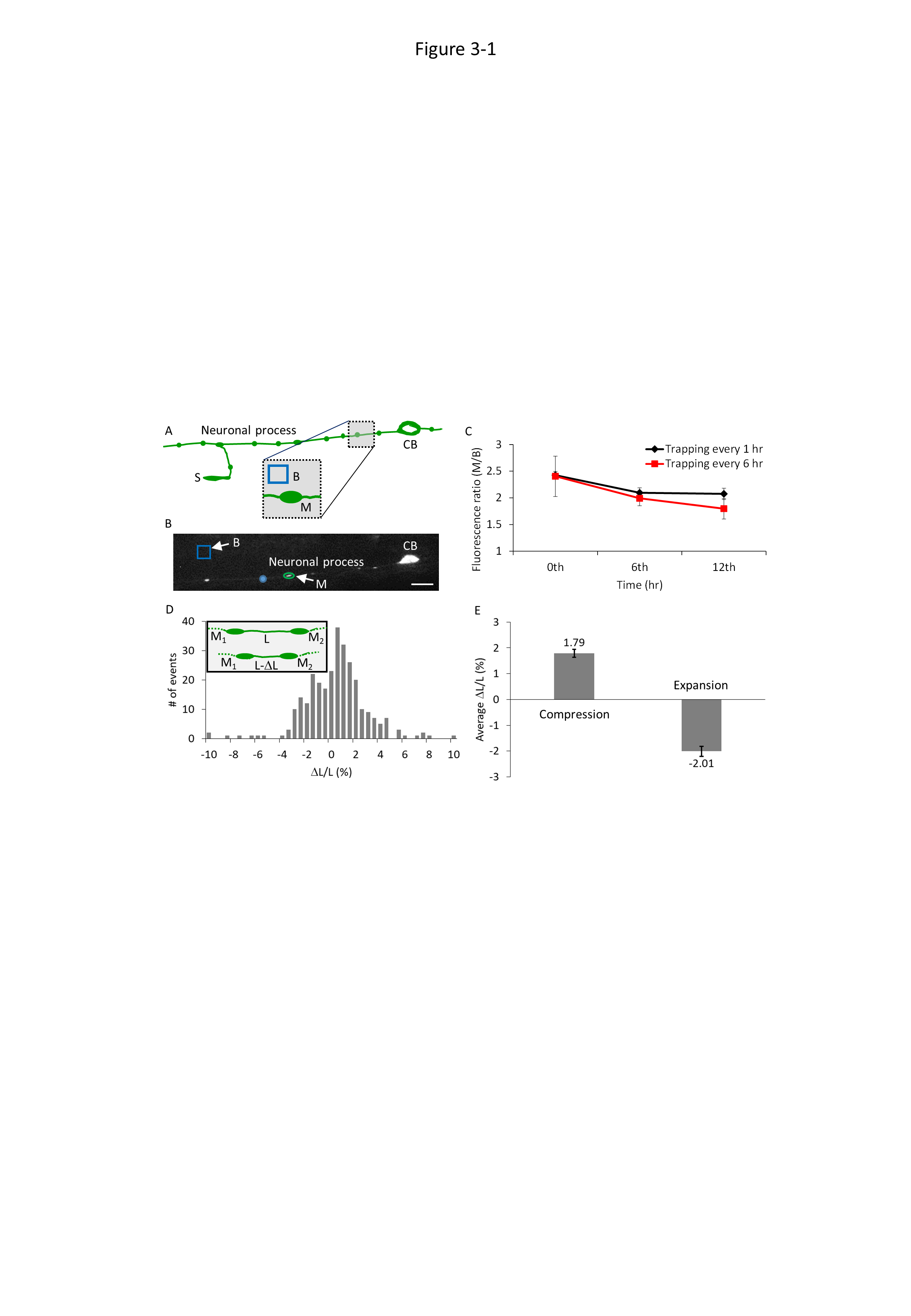

Supplement: Extended Data Figure 3-1 — Effect of photobleaching and intermitochondrial distances during long-term mitochondrial imaging. A, Schematic of the TRN neuron. The inset shows a single mitochondrion and a 5 × 5 μm box to calculate the average intensity values for a mitochondrion (M) and the background (B). B, Image of a single neuron at zero time shows a single mitochondrion (M) and the background box (B) on the worm body. Scale bar: 10 μm. C, Fluorescence ratio of the mitochondria intensity to background intensity (M/B) calculated from the images of the same animal captured at 0th, 6th, and 12th hour imaging time points. The animals were immobilized at two different time intervals of every 1 h and every 6 h, respectively. Time-lapse imaging of the same animal over 12 h shows statistically insignificant photobleaching of mitochondrial fluorescence (n = 4 animals, 5 mitochondria, and a box drawn around each mitochondrion was used to calculate the statistics). Data represented as mean ± SD. Statistical significance was evaluated by one-way ANOVA with Bonferroni post hoc comparisons; nonsignificant values (p > 0.05) are not indicated. D, Number of events for the percentage of compression or expansion measured from relative intermitochondrial distances (ΔL/L, n = 292 events). The inset shows a pair of stationary mitochondria (M1 and M2) with an intermitochondrial distance of L compressed to L-ΔL. E, Average compression (M1 and M2 appear closer) or expansion (M1 and M2 move further apart) percentage values. The data represented as mean ± SEM (n = 8 animals imaged for 3 successive time points at 5 min intervals). Download Figure 3-1, TIF file. [file enu-eN-NWR-0360-20-s05.tif]

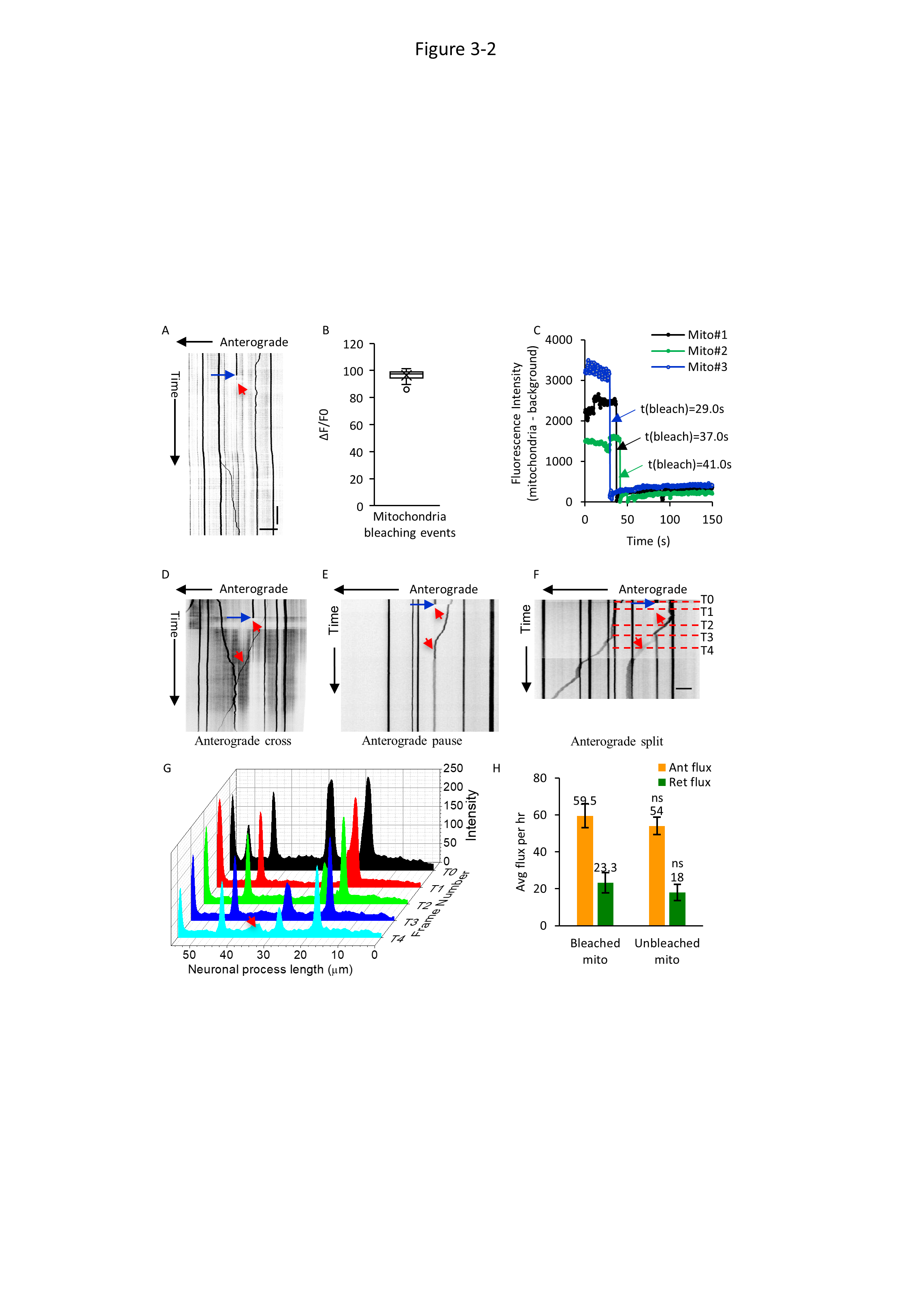

Supplement: Extended Data Figure 3-2 — Kymographs of levamisole immobilized L4 C. elegans that show bleaching of stationary mitochondria and anterogradely moving mitochondria across the bleached mitochondria. A, Kymograph of stationary mitochondria (red up arrow) that was bleached at 41.0 s (horizontal blue arrow) in the PLM neuronal process. Horizontal and vertical scale bars: 20 μm and 33 ms, respectively. B, The box and whisker plot shows the percentage drop in the integrated mitochondrial fluorescence intensity during photobleaching of n = 20 stationary mitochondria. The plot shows the median line, mean marker (×), and the outlier (o). C, The fluorescence intensity profile of stationary mitochondria that was bleached using a 405-nm laser. The arrow indicates the time when each of the three mitochondria were bleached at 29.0, 37.0, and 41.0 s. D–F, Anterogradely moving mitochondria (red down arrow) crosses (D), pauses (E), and partially splits (F) at the site of the bleached mitochondria (red up arrow). The blue horizontal line indicates the bleaching of the stationary mitochondrion. Scale bar: 10 μm. G, The intensity distributions along the neuronal process length and at five different time points (T0, T1, T2, T3, and T4), represented by the red dotted lines in F. H, The average flux of moving mitochondria across a bleached (n = 15) and an unbleached (n = 18) mitochondrion. The data represented as mean ± SEM. Statistical significance was evaluated by paired sample t test; nonsignificant values (ns, p > 0.05) are represented. Download Figure 3-2, TIF file. [file enu-eN-NWR-0360-20-s06.tif]

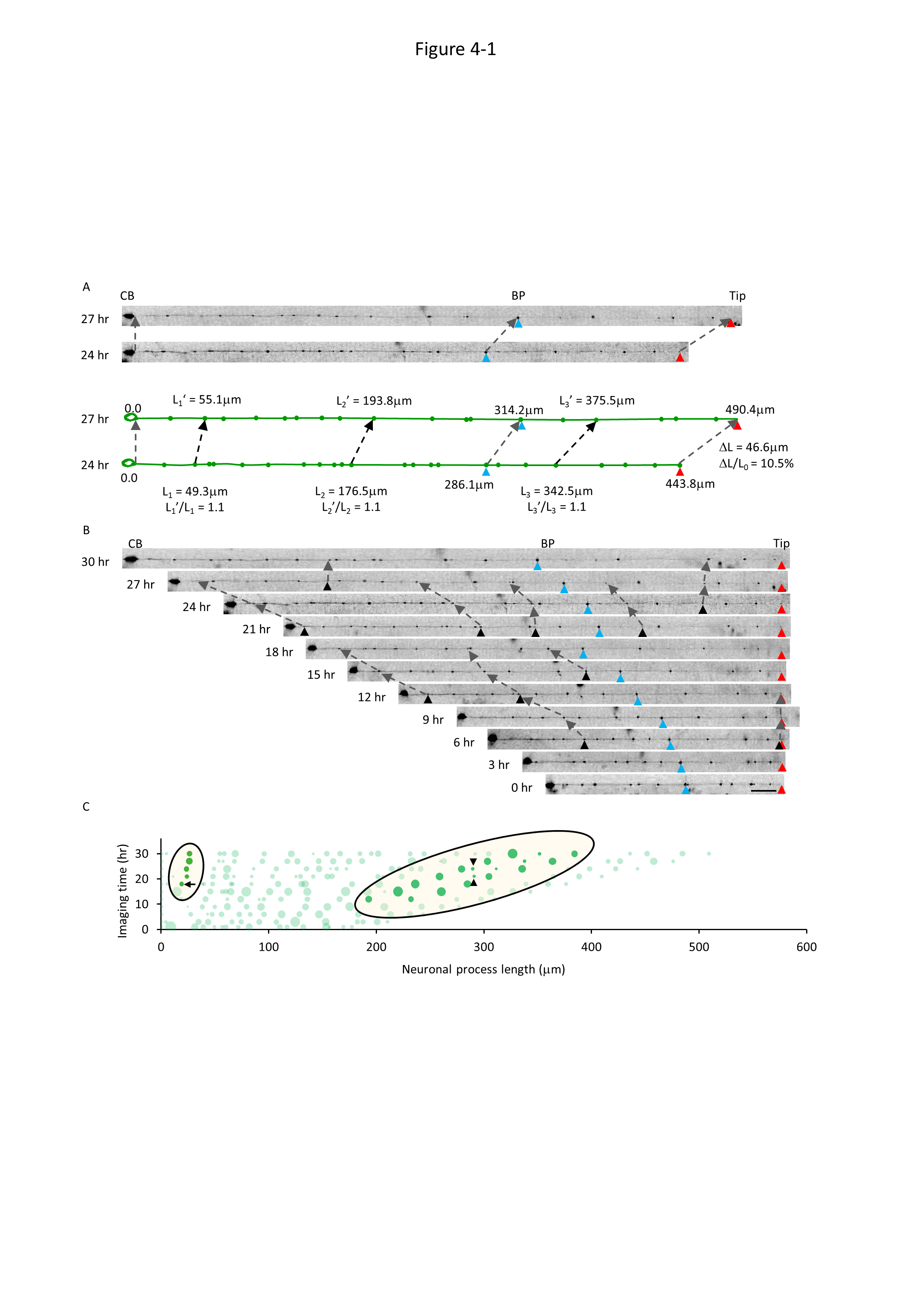

Supplement: Extended Data Figure 4-1 — Time-lapse images of the neuron from an individual animal. A, The images of the entire neuronal process are shown at 24- and 27-h time points after start of imaging. Two images are aligned with respect to the cell body (CB) and the two additional reference points- the BP (blue triangle) and neuronal end (Tip; red triangle) are marked on the images. The CB, BP, Tip are connected with gray dotted arrows. The trace below the images represents the neuronal processes for both time points with the mitochondria indicated as green dots. The position of fiduciary markers (CB, BP, and neuronal Tip) and three mitochondria are indicated at time t =24 h (L1, L2, and L3) and t =27 h (L1’, L2’, and L3’) and connected using gray dotted arrows. The ratio of the two distance between given mitochondria from the CB at 24 and 27 h corresponds to the observed 10% (46.6 μm) increase in total neuronal process growth (ΔL = 46.6 μm) in this 3-h period over the initial process length of L0 = 443.8 μm at t = 24 h. B, Alignment of the neuronal process image of Figure 4F for all 11 time points at the tip. C, A representative bubble plot of all 11 time points with the diameter of the bubble proportionate to the total area of each mitochondrion. The highlighted region shows the addition of a new smaller mitochondrion (black arrow and black triangles) between two identified adjacent mitochondria. The small mitochondrion added on 18th hour (black arrow) increases in size in successive imaging frames. A small mitochondrion added at the 24th hour (black inverted triangle) did not persist over 6 h and was not considered as a mitochondrion addition event. Download Figure 4-1, TIF file. [file enu-eN-NWR-0360-20-s07.tif]

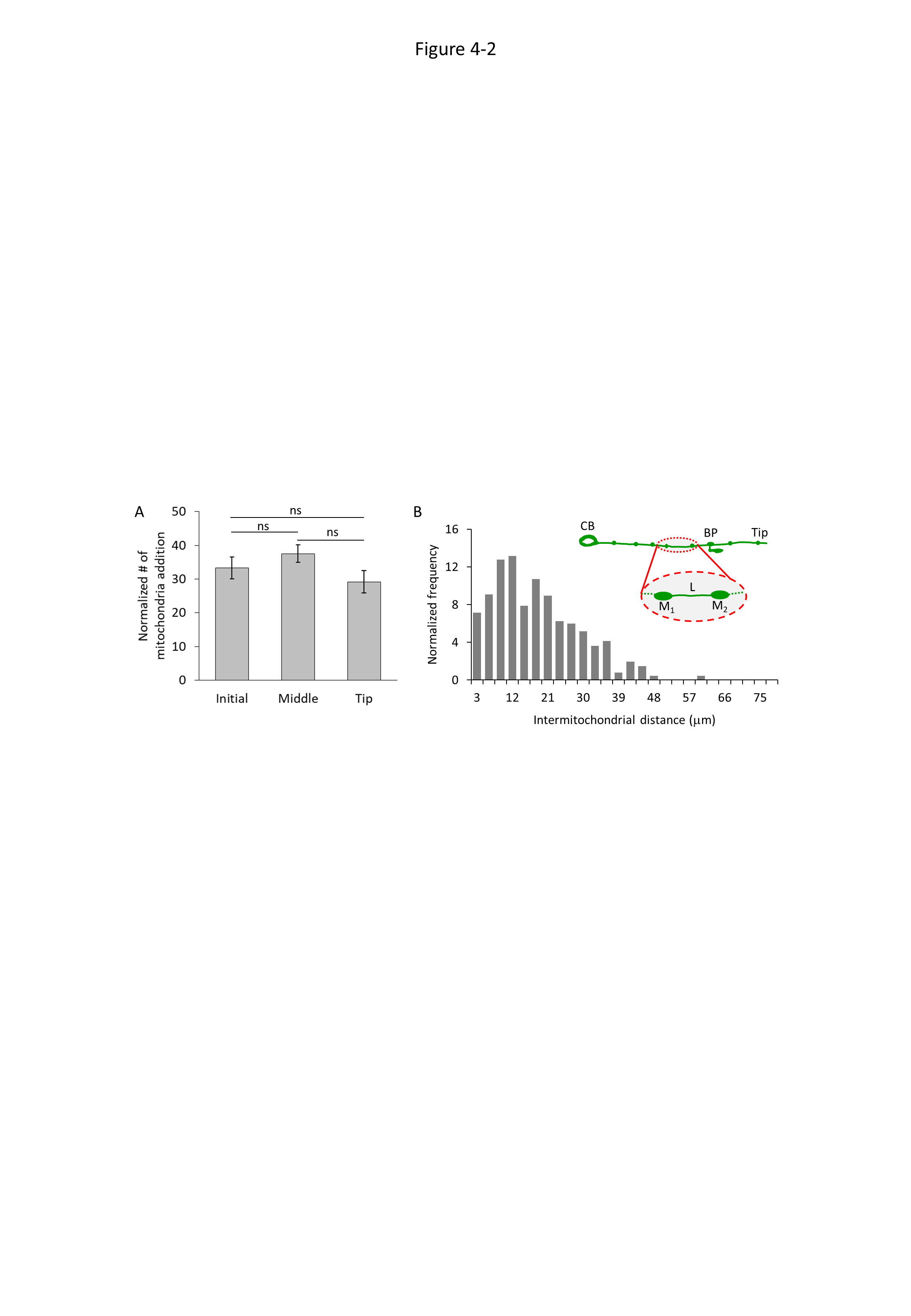

Supplement: Extended Data Figure 4-2 — Mitochondria addition events and intermitochondrial distances measured along the neuronal processes. A, The normalized number of mitochondria added along each segment of the neuron where each segment corresponds to an equal third of the neuronal process. The total number of mitochondria addition events are 85 (9, 10, 10, 10, 11, 15, 14, and 6 for each of the eight animals imaged). Data represented as mean ± SEM. Data were compared using one-way ANOVA with post hoc Tukey correction for multiple comparisons (ns indicates nonsignificant for p > 0.05). B, Normalized number of intermitochondrial distances in L4 stage animals (n = 8 animals) immobilized in the microfluidic chip. The schematic of the mechanosensory neuron with cell body (CB), BP, and neuronal end (Tip). The inset shows a pair of mitochondria (M1 and M2) with an intermitochondrial distance L. Download Figure 4-2, TIF file. [file enu-eN-NWR-0360-20-s08.tif]

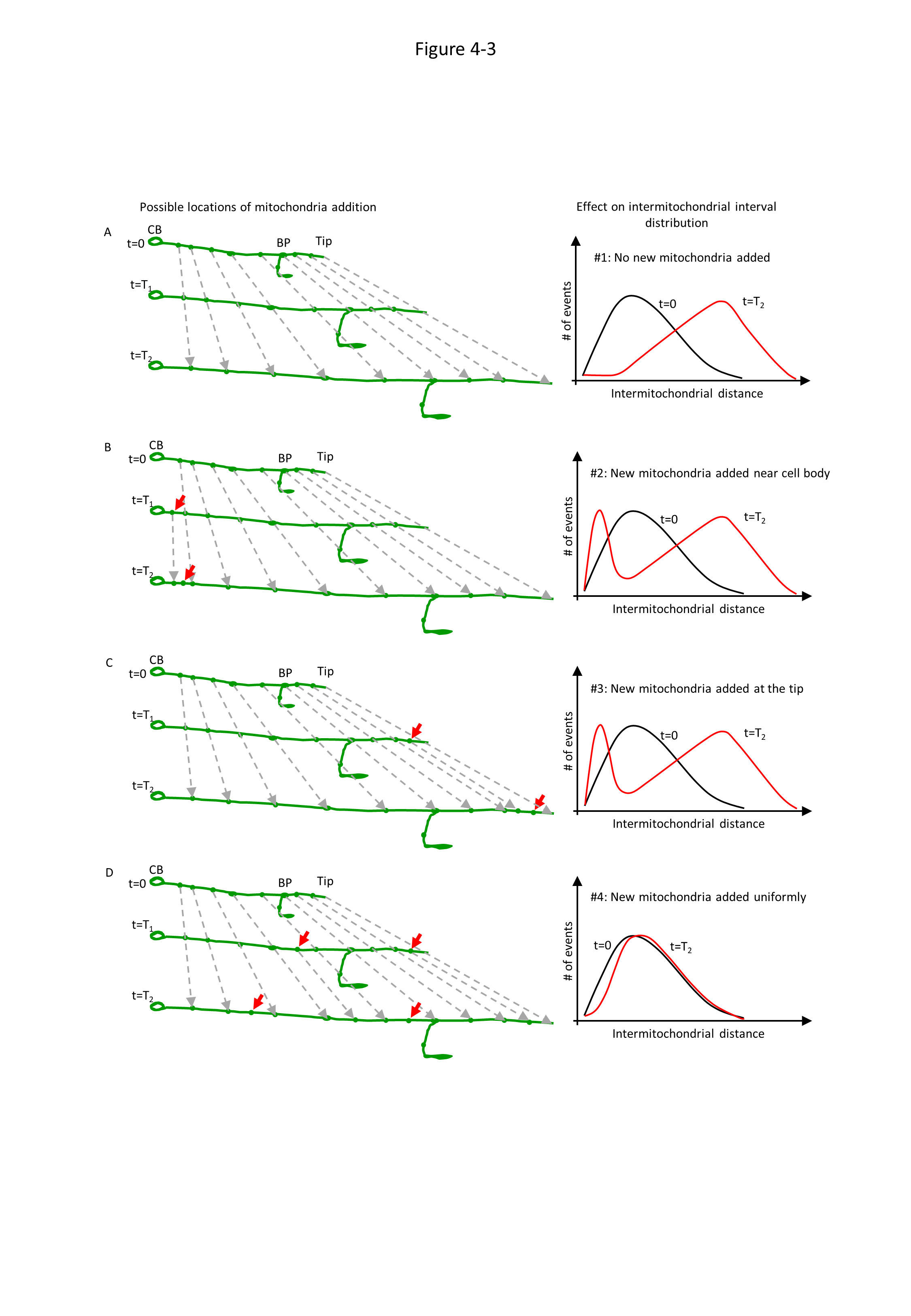

Supplement: Extended Data Figure 4-3 — Schematic representation of mitochondria addition events between preexisting adjacent mitochondria in a growing neuronal process and the corresponding changes in intermitochondrial intervals after mitochondria additions. No new mitochondria added (A), new mitochondria added near the cell body (B), new mitochondria added at the end of the neuron (C), and new mitochondria added uniformly along the neuronal process (D). The addition of a new mitochondrion occurs when the intermitochondrial distance between adjacent mitochondria increases beyond a threshold. Three different time points are denoted that are sequentially (t = 0, t = T1, and t = T2). The red arrow points to newly added mitochondria. The dashed arrows are added to highlight the position of each stationary mitochondrion in successive time points. The panel on the right is a schematic representation of the expected change in intermitochondrial distances for each possible addition paradigm. Download Figure 4-3, TIF file. [file enu-eN-NWR-0360-20-s09.tif]

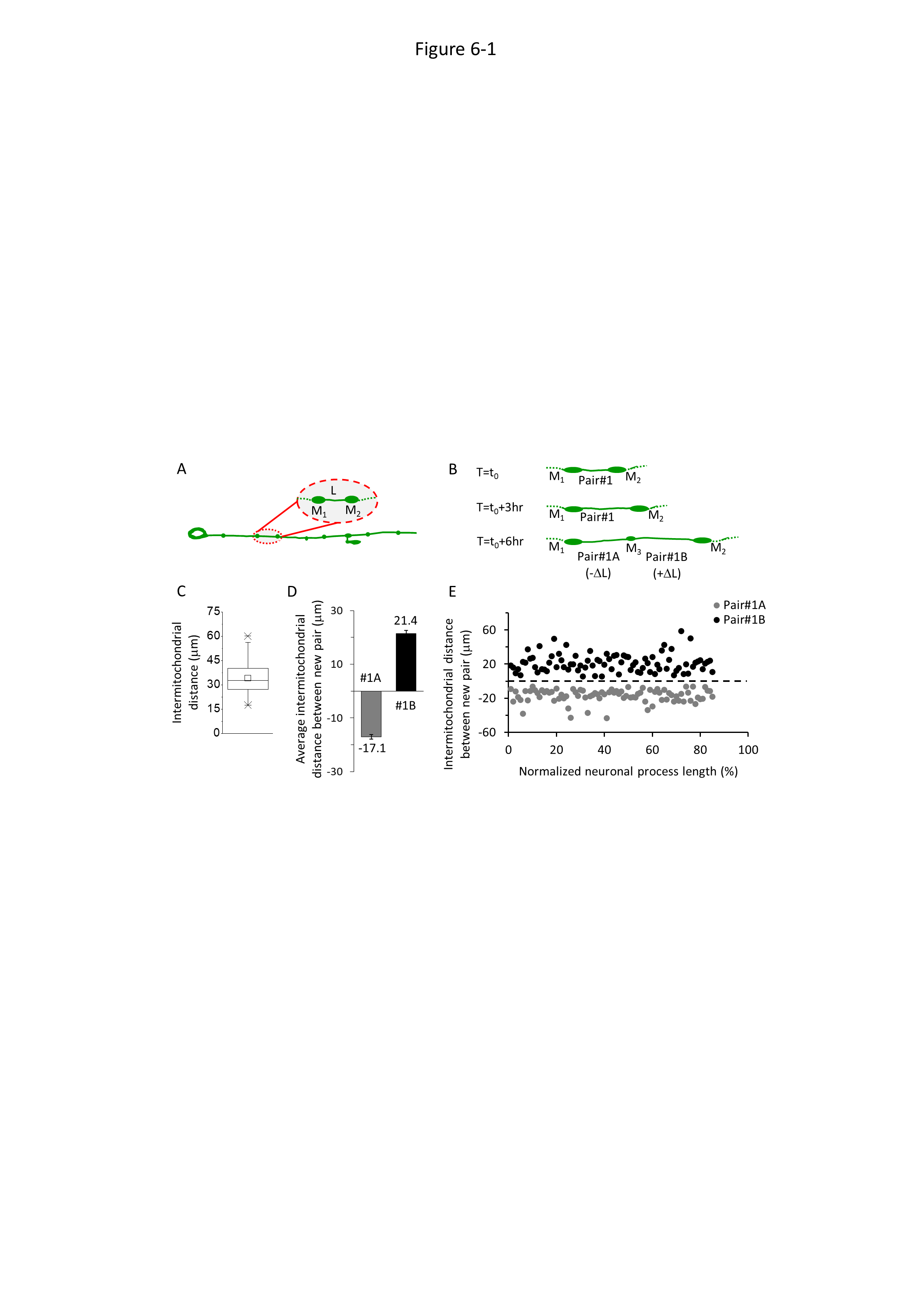

Supplement: Extended Data Figure 6-1 — Intermitochondrial distance statistics when new mitochondria are added between a pair of adjacent mitochondria. A, Schematic of a mechanosensory neuron with the inset highlighting an adjacent mitochondrial pair along the neuronal process. B, Schematic of the neuronal process that shows the increase in the intermitochondrial distance between the pair of mitochondria at three different time points (t0, t0 + 3 h, and t0 + 6 h). A new mitochondrion is added at t0 + 6 h. The intermitochondrial distance is measured between the old (pair #1) and new adjacent pairs (pairs #1A and #1B) of mitochondria at time points before and after the addition of the new mitochondrion, respectively. C, Box and whisker plot of all the intermitochondrial distances from time-lapse imaging of the neuronal processes of animals (n = 8) grown inside the microfluidic chip and imaged for 36 h. The box represents 25th and 75th percentiles, whiskers represent outliers, and the center small box represents the mean (n = 85 mitochondrial pairs). D, The bar graph of intermitochondrial distances between new pairs of adjacent mitochondria after the addition of a new mitochondrion between a pair of old adjacent mitochondria (n = 85 new pairs). E, The intermitochondrial distance for all 85 new pairs (pairs #1A and #1B) are plotted as a function of the normalized neuronal process location, where an addition event occurred. Download Figure 6-1, TIF file. [file enu-eN-NWR-0360-20-s10.tif]
